# Causal Inference Meets Deep Learning: A Comprehensive Survey

**DOI:** 10.34133/research.0467

**Published:** 2024-09-10

**Authors:** Licheng Jiao, Yuhan Wang, Xu Liu, Lingling Li, Fang Liu, Wenping Ma, Yuwei Guo, Puhua Chen, Shuyuan Yang, Biao Hou

**Affiliations:** The School of Artificial Intelligence, Xidian University, Xi’an, China.

## Abstract

Deep learning relies on learning from extensive data to generate prediction results. This approach may inadvertently capture spurious correlations within the data, leading to models that lack interpretability and robustness. Researchers have developed more profound and stable causal inference methods based on cognitive neuroscience. By replacing the correlation model with a stable and interpretable causal model, it is possible to mitigate the misleading nature of spurious correlations and overcome the limitations of model calculations. In this survey, we provide a comprehensive and structured review of causal inference methods in deep learning. Brain-like inference ideas are discussed from a brain-inspired perspective, and the basic concepts of causal learning are introduced. The article describes the integration of causal inference with traditional deep learning algorithms and illustrates its application to large model tasks as well as specific modalities in deep learning. The current limitations of causal inference and future research directions are discussed. Moreover, the commonly used benchmark datasets and the corresponding download links are summarized.

## Introduction

As a crucial research direction in artificial intelligence (AI), deep learning has demonstrated a broad spectrum of applications and impressive performance in numerous fields, including visual learning [1–4], natural language processing (NLP) [5,6], medical research [7–9], speech recognition [10,11], machine translation [12], video understanding [13,14], biological research [15,16], and others. In contemporary deep learning methodologies, predictive outcomes are acquired through extensive data assimilation. However, this learning approach is susceptible to learning spurious correlations within the data, thereby disregarding intrinsic causal relationships. Consequently, the accuracy and robustness of the model’s discernment are compromised, impeding its ability to generalize across different domains. Current methodologies leverage self-supervised learning [17], semisupervised learning [18,19], and reinforcement learning [20,21] to enhance model robustness. These approaches predominantly focus on data-centric model acquisition, relying on extensive labeled data or interaction volume with the environment to achieve satisfactory performance. We advocate for models that comprehend the causal framework underlying the data, transcending mere correlation. This approach facilitates better outcome prediction and adaptation to data distributions in new and unexplored domains. With the development of deep learning, the requirements for algorithms not only are fast and accurate in prediction but also expect the models to be interpretable, trustworthy, and robust. Traditional models, trained on independent and identically distributed (IID) data, confront performance degradation when navigating intricate and evolving environments [22], particularly in domains demanding heightened robustness and adaptability like medicine and autonomous driving [23,24]. Therefore, researchers have begun to explore the construction of deeper and more stable causal relationships and apply them to various areas of deep learning to improve the performance of models in dynamic environments.

### Overview and organization

Causal learning serves to identify and mitigate data biases and spurious associations [25], thereby enhancing the robustness of models. A further understanding of deep learning models can be achieved by examining them through the perspective of causal learning, providing valuable insights to address weaknesses and anticipate potential issues. This approach enhances the robustness of the model and improves its performance across various tasks [26,27].

Causality research can generally be divided into 2 primary branches [28,29]: causal discovery and causal inference. Causal discovery explores the relationship between data by identifying the causal relationships between variables based on observed data and determining which variables can be influenced by changes in another variable. This process involves statistical analyses, machine learning techniques, or computational methodologies to uncover causal structures within data. Causal discovery is primarily utilized to investigate unknown causal structures [30]. Existing methods rely on a substantial amount of data and computational resources, which can be challenging to obtain in practical applications. Therefore, although this method has very important applications in scientific research, it has relatively little practical use in deep learning. Causal inference aims to study causal effects and assess the influence of a causal factor on another variable or outcome [31]. It involves quantifying the strength of the causal relationship between the cause and effect, assuming that a causal structure is known to exist between the 2. This approach enables the design of rational models to estimate causal effects using available observational data. In deep learning tasks, we typically investigate the influence of causal effects on the outcomes assuming the presence of causal relationships within the data. Therefore, this paper will delve further into the description of causal inference.

The analysis and reasoning of causal relationships play a critical role in scientific research as they are often considered the fundamental goal of scientific inquiry. However, in the previous deep learning field, causal inference has not yet been given the same level of importance. As deep learning continues to advance, data-driven learning is facing increasingly complex challenges. As a mathematization of human thinking [32], causal inference has been gradually applied to the development of various modeling algorithms because of its low dependence on data and high generalizability [33]. In recent years, causal inference has found increasing applications in various fields of deep learning, such as visual representation [34], video processing [13,35,36], visual-linguistic analysis [37,38], interpretability of deep learning [39,40], and NLP [41]. These applications have enabled the construction of models through deep causality, thereby facilitating the process of inferential and cognitive tasks.

**Fig. 1.**
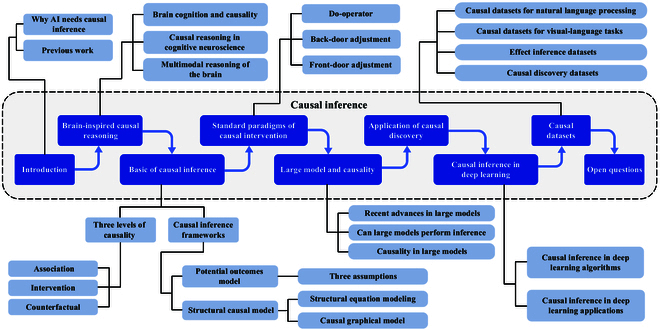
An overview of the survey.

In this paper, we introduce the basic concepts of causal inference and explore the ideology of brain-inspired reasoning. We review the theoretical foundations and general frameworks used in causal inference, and summarize its integration with classical algorithms for deep learning. Additionally, we systematically describe the cross-applications of causal inference in various areas of deep learning, highlighting the research implications of causal inference in real-world tasks. Finally, we provide insights into commonly used datasets for causal tasks, along with anticipated challenges that may arise in future research. An overview of our paper is shown in Fig. 1. The specific contributions are outlined as follows:

• The fundamental concepts of causal inference from a brain-inspired perspective are introduced in this paper. Two foundational frameworks for causal inference, the potential outcomes model (POM) and the structural causal model (SCM), are explored, and a comparative analysis of their interconnections is provided.

• The latest advancements in large-scale models are introduced, and causal inference within large language models (LLMs) is analyzed.

• The integration of causal inference with classical algorithms for deep learning is described. Additionally, the cross-applications of causal inference in different areas of deep learning are systematically described, with a special emphasis on its application in visual representations, and the research implications of causal inference in real-world tasks are illustrated.

• Summarizes natural language as well as vision-based causal datasets for exploring causal relationships in data. The limitations of causal inference and future research directions are discussed.

The content framework of this paper is illustrated in Fig. 1. In the “Brain-Inspired Reasoning” section, brain-inspired causal reasoning is discussed. In the “Basic of Causal Inference” section, the 3 levels of causality, along with the 2 common causal inference frameworks and their connections, are introduced. In the “Standard Paradigms of Causal Intervention” section, we introduce the symbolic representations of causal interventions, as well as the concepts and formulas of the front-door criterion and back-door criterion. In the “Large Model and Causality” section, inference capabilities in LLMs are analyzed. In the “Application of Causal Discovery” section, relevant applications of causal discovery in AI are described. In the “Deep Learning Algorithms with Causal Inference” section, the combination of causal inference with classical algorithms for deep learning is presented. In the “Deep Learning Applications with Causal Inference” section, we focus on the integration of causal inference with various areas of deep learning, detailing the application of causal inference to 4 specific modalities: voice (speech processing), text (NLP), graphics (graph representation learning), and images (visual representation). In the “Causal Datasets” section, some causal-based visual and linguistic datasets are introduced. Finally, in the “Open Questions” section, the prospects and limitations of causal inference and the top 10 open questions are presented.

### Why AI needs causal inference

The reasons we believe that AI requires causal inference include the following:

#### Improved accuracy of decision-making

Existing models predict outcomes based on data correlation. While the predicted results may meet accuracy requirements, these models cannot provide explanations for their decisions [42]. Consequently, machines are unable to solve problems like humans. Particularly in domains requiring low fault tolerance, such as healthcare and transportation, machines cannot completely replace human intervention.

The importance of causal inference lies in its capacity to assist systems in gaining a better understanding of the relationships between events, discerning the causes and effects of events rather than solely focusing on the correlations between variables. For instance, in the context of autonomous driving tasks, when faced with accidents during autonomous driving tests, a model may analyze various variables contributing to the occurrence of accidents, such as road conditions, vehicle speed, and positions of other objects, and identify their correlations. Incorporating causal inference can assist the system identify which variables cause accidents, which variables change concurrently with accidents, and the extent to which different variables impact accidents. This approach can help enhance the safety of autonomous driving systems. In practical applications, causal inference can help AI systems better understand the true causal relationships between events. Samsami et al. [43] used causal inference methods to address 2 undesirable behaviors that arise from neglecting the causal structure of expert driving demonstration data: inertia and collisions. Ding et al. [44] introduced causal relationships into the scene generation process to enhance the robustness of autonomous driving algorithms. These methods can improve the accuracy of system decisions and more accurately predict the outcomes of actions.

#### Improving model generalization and robustness

We analyzed various tasks based on deep learning and found that existing methods may face challenges such as poor generalization ability and low robustness [45], leading to reduced predictive performance in complex and novel datasets. The main reason for these issues is that most current learning models rely heavily on correlations between data rather than learning the causality within the data. Consequently, they are susceptible to being misled by differences between data samples, thereby affecting the robustness of the model [46]. For instance, the autonomous driving model we trained using the Chinese road traffic dataset would get worse results if applied to UK roads. This is due to the difference in driving rules: Cars drive on the right side in China and on the left side in the UK. This difference has forced us to retrain new AI models. While AI still has significant limitations, we hope that models can explain the relationship between variables that arise in the environment and respond to changes in those variables [33]. The emergence of causal inference models highlights the causal mechanisms in the data and provides a path to address these issues. Sui et al. [47] employed causal assumptions and proposed a causal attention learning strategy to construct causal patterns, thus alleviating the confounding effects of shortcuts. Sun et al. [48] introduced latent variables to differentiate genuine causal factors from spurious correlated variables, employing causal relationships in domain generalization tasks. These methods substitute stable and interpretable causal models for associative models, reducing the misleading effects of spurious correlations and overcoming limitations in model computations.

#### Improving the interpretability of models

The interpretability [49] of models holds significant importance. Interpretable models can provide reasonable explanations for their decisions, making it easier for users to comprehend the reasons behind these decisions. This enhances model credibility and assists developers in identifying errors and biases within the model. With the continuous research on interpretability in machine learning, researchers have proposed many interpretable machine learning methods, including rule-based models [50], local surrogate models [51,52], and more. With the emergence of causal learning, increasingly causal-oriented methods have appeared in interpretable learning. Causal inference can help construct causal graphs to clearly present the causal relationships between variables, facilitating the understanding of how models make predictions. Some causal models are specifically designed to enhance model interpretability, evaluating the impact of different scenarios on prediction results through interventions or counterfactual methods, thereby helping users understand the importance of changing different variables on decision outcomes. For example, Xu et al. [53] proposed a method based on causal inference to understand the inherent mechanisms of complex models. This approach helps explain model behavior and operational mechanisms, thereby enhancing model interpretability. Wu et al. [54] introduced an efficient new paradigm for modeling from a causal perspective, designing neural interpreters using appropriate causal graphs and important causal principles to enhance model performance.

### Previous work

In order to provide the reader with an understanding of research on causal theory from various perspectives, we summarize and discuss the majority of the existing comprehensive surveys based on causal learning. As a classical comprehensive survey of causal learning, Pearl [55] aimed to introduce the latest advances in causal inference based on SCMs, and provided 3 causal assessment mathematical tools in the causal domain: potential outcomes, counterfactuals, and direct and indirect effects. Several researchers have explored the relationship between causal discovery and machine learning using various types of data. Guo et al. [56] described methods for learning causal effects (causal inference) and causal relationships (causal discovery) from different data types, and how the causal discovery problem relates to machine learning. Moraffah et al. [57] investigated 2 main tasks for time series data using causal inference methods: effect estimation and causal discovery tasks for time series data. Chen et al. [58] categorized causal discovery tasks into 3 types from the perspective of variable paradigms. Liang et al. [59] discussed the potential for causal analysis in the AI field and the challenges of algorithmic convergence. The article provides a concise overview of the theoretical advancements and practical applications of causal analysis across diverse domains, including neuroscience, quantum mechanics, finance, and beyond. Other researchers have made comprehensive assessments of causal inference methods in the fields of statistical learning and machine learning, with the goal of investigating causal effects between variables. Yao et al. [60] provided a comprehensive assessment of causal inference methods for potential outcome frameworks based on traditional statistical and machine learning methods. Schölkopf [61] emphasized the connection between graphical causal inference and machine learning and AI. Lu [62] proposed a comprehensive causal representation learning framework that addresses generalization issues based on assumptions in causal relationships. Additionally, it solved the adaptation problems required by reinforcement learning by constructing concise image representations.

Most of the existing work focuses on causal discovery tasks, traditional statistical learning, and machine learning but lacks a comprehensive integration of causal learning in the field of deep learning. Luo et al. [63] introduced several research methods that provide model interpretation by encoding causal relationships between random variables. For example, causal inference is parameterized by constructing a linear structural equation model, thus transforming the original problem into a continuous optimization problem [64], or by using Bayesian networks (BNs) and directed acyclic graphs (DAGs) to extend to nonparametric problems [65,66]. Although methods for causal inference using deep neural networks (DNNs) are described, they are understudied. Berrevoets et al. [67] introduced the concept of causal deep learning (CDL), described a practical framework for CDL in terms of structural, parameter, and temporal dimensions, and provided examples of applying CDL in the fields of healthcare, economics and business, environmental science, and education. Zhou et al. [68] gave a structured approach to comprehending the mechanism of data generation through causality. The exploration delves into the intersection of causality and deep generative models (DGMs), covering various aspects from fundamental principles to specific applications. It also discusses how LLMs perform the task of causal inference. However, its research focuses solely on understanding and modeling data generative processes (DGPs). Kaddour et al. [69] focused on causal machine learning (CausalML), a machine learning approach that formalizes the data generation process into SCMs. This approach is categorized into 5 areas such as causal supervised learning, causal generative modeling, causal interpretation, causal fairness, and causal reinforcement learning. It systematically compares the methods and outlines future research directions within each category. This article provides a detailed categorization and comparison of current CausalML research. However, it lacks specificity and comprehensiveness for the practical application of causal learning in specialized domains like computer vision, NLP, and graph representation learning. Liu et al. [34] reviewed existing causal inference methods for visual representation learning, including basic theories, models, and datasets of causal learning. Although the study aimed to explore visual representation learning based on causal inference, the presentation is not comprehensive enough and lacks examples of causal-based visual tasks. Feder et al. [70] integrated causality research across interdisciplinary fields and explored the problem of estimating causal effects in the broader field of NLP. However, it focuses more on the field of NLP, and the scope of deep learning involved is not broad enough.

In comparison with these studies, our comprehensive survey has the following innovations: (a) The idea of causal inference is explored from a brain-inspired perspective. (b) The reasoning power of large models and their contribution to causal learning is discussed. (c) We introduce the combination of causal inference methods with traditional deep learning algorithms. Importantly, we provide a more comprehensive categorization and a richer novelty in the cross-over study of causal inference and deep learning tasks involving various data types. (d) We have summarized the proposed models, publication dates, causal task classifications, and key attributes of all related work in a tabular form to facilitate readers’ access. (e) A more comprehensive summary of causal datasets is provided, including their corresponding download links.

## Brain-Inspired Reasoning

The brain is a complex and intelligent system [71,72]. Today’s AI is based on the inductive logic of the human brain. However, the problems in human society are complex and diverse, and not all conditions in the world can be realized. Most events cannot be mathematically modeled based on actual conditions, which is a limitation of traditional AI. By drawing inspiration from brain cognition and neuroscience, we can create a more advanced and sophisticated form of AI that can better replicate the intricacies of the human brain. While current AI technology has made tremendous strides in terms of computational power, it still falls short when it comes to replicating the complex and adaptable nature of human thinking. This is particularly true in situations where events are open-ended, constantly changing, and highly complex. In these scenarios, the human brain is able to reason and make sense of the situation, often uncovering unknown information from known clues. This is a challenge that AI must overcome if it is to fully replace human intelligence. Therefore, the development of AI that is closer to the human brain is critical for unlocking the full potential of AI and realizing its transformative power.

### Brain cognition and causality

Causality has been studied in various fields, including philosophy, psychology, and statistics, each placing different emphases on its significance. From the perspective of brain cognition, causality represents a regularity between cause and effect [73]. In real society, how do humans make judgments about causality? On one hand, they rely on established logical rules. on the other hand, they rely on a priori knowledge.

In brain neuroscience, how does the brain make causal reasoning? The complex regions and structures of the brain make it very challenging to judge the correspondence between different brain regions and their functions [74]. Luria et al. [75] proposed the functional block theory of the brain, which suggests that the brain can be divided into 3 main units responsible for alertness, perceptual reception and integration, and the planning and execution of behavior. Cognitive psychologist Evans [76] introduced the dual-process theory, which explains the existence of 2 distinct cognitive thinking systems in the human brain. These systems are heuristic intuitive judgment and systematic rational analysis. Kuo et al. [77] conducted experiments that demonstrated the reliance of the human brain on different parts to process 2 tasks, intuition and reasoning. He argued that the human brain has 2 neural mechanisms: intuition (fast and emotional) and reasoning (slow and controlled). According to dual-process theory, researchers have categorized reasoning into intuitive reasoning and logical reasoning. Logical reasoning is based on causal logic and data analysis, exploring complex cause-and-effect relationships in depth. Intuitive reasoning is based on knowledge and a priori. It is considered the ability to quickly determine answers based on intuition and previous experience. Intuitive reasoning in the human brain relies on cognitive models constructed in advance based on environmental relationships.

O’Keefe and Dostrovsky’s [78] experiments were the first to identify positional neurons in the mammalian brain by studying brain activity in rats. These experiments led researchers to propose that the hippocampus, a region in the mammalian brain, is capable of creating “cognitive maps” [79,80]. Cognitive maps are interpretable cognitive models constructed in the brain based on a priori knowledge. They are mental representations of the environment and spatial relationships in the brain. It can help individuals navigate and understand spatial relationships, as well as help organisms in learning and interpreting environmental information [81]. As humans learn and accumulate experiences, they continue to refine their cognitive maps and receive feedback through dopamine rewards, which further improves the computational model of the brain. This enables them to make inferences and decisions based on the maps they have constructed.

### Causal reasoning in cognitive neuroscience

Research in brain neuroscience has shown that thinking is a complex process that involves multiple regions of the brain working together. Damage to different parts of the brain can have varying effects on thinking, behavior, and emotion. Some neurobiologists have suggested that the interaction between neurons in the brain is responsible for these processes, with synaptic connections facilitating the processing and transmission of information. Studies have also revealed that neuronal populations are capable of accurately encoding causal relationships from a small amount of information, highlighting the remarkable efficiency of the human brain for causal understanding. This ability for causal encoding is both a basic process, such as human perception, and a complex process, such as decision-making for causal inference [82,83]. Researchers have analyzed the causal structure of the neural system using random graphs with arbitrary structures, instead of artificial designs. Neurons do not respond to all stimuli, and there are complex relationships between different neurons that are difficult to judge independently.

Simple behaviors can rely on relatively direct interactions between brain systems, while more complex behaviors require the involvement of a large number of neurons (the human brain contains 100 billion or more). The richness of external information carried by this vast population of neurons is also accompanied by interference and confusion [84]. To extract useful information from this immense volume and reduce confusion, the human brain has developed an important mechanism to coordinate lower-level sensory and motor processes to handle cognitive tasks and causal thinking in the presence of complex information. This cognitive ability involves multiple regions within the brain. However, Miller et al. [84] suggested that the prefrontal cortex (PFC), which possesses high-level cognitive functions, plays a decisive role. The PFC connects and coordinates neural processes across multiple regions to manage cognitive control and goal-directed thinking.

A large population of neurons in the PFC is capable of working with small amounts of information to achieve high-accuracy causal encoding, demonstrating the efficiency of the human brain in causal understanding. In addition, the PFC possesses a working memory function that plays a crucial role in causal reasoning. Working memory can maintain information relevant to the task at hand during the execution of cognitive tasks, providing temporary storage and enabling the manipulation and processing of information [85]. It filters and controls important information in complex tasks, serving as a crucial mechanism in the execution process. It aids the brain in conducting causal analysis and logical reasoning under complex informational conditions. Most importantly, the PFC also has the ability to control attention. Desimone et al. [86] suggested that information processing in the brain is competitive, with different pathways carrying different information competing for behavioral performance, and information with strong support prevailing. During this process, neurons responsible for processing various perceptions interact and compete with each other, with the winning neuron becoming more active. The PFC generates a top-down excitatory signal that represents the features of the scene requiring attention. This signal creates a biased selection in the competition between neurons, assisting in the shifting of attention. This bias signaling is equivalent to real-time judgment and management of causality, considering various information inputs and historical experiences to determine the most appropriate behavioral action. This mechanism helps the brain selectively focus on relevant information and ignore useless and confusing information, enabling it to judge causality in complex environments and reason about subsequent behavior. Asaad et al. [87] argued that complex behavior in the brain relies on more intricate rule mappings rather than simple eventualities. Therefore, causal reasoning for realistic tasks also focuses on the judgment of causal rules. Brain mechanisms in cognitive neuroscience are studied to help machines understand and model cognitive functions. These mechanisms are simulated in terms of cognitive encoding of information, memory storage, and attentional control to construct a robust model of causal reasoning.

### Multimodal reasoning of the brain

In addition, when humans need to judge different sensory information, they rely on the reliability of different senses, which is known as reliability weighting [88]. Imagine a mosquito that keeps buzzing around you and finally lands on your arm [89]. At this point, you have 2 senses to rely on for determining where to swat: visual and sensory. Mathematically, these 2 senses are combined to judge the position of the mosquito, with the more reliable and less error-prone one being assigned greater weight. Extensive research [90] has shown that for multimodal matching tasks, human behavior aligns with the mathematical logic of integrating multiple perceptions. However, when the 2 sensations come from different locations, our brain needs to make a judgment. Under the premise that visual information is more reliable, the itchy sensation may originate from other factors, such as previous mosquito bites. In such cases, we need to ignore the tactile sensation and base the next action solely on the feedback provided by visual information. This step involves our brain engaging in “causal inference” to determine whether the 2 pieces of information are from the same source and if they need to be integrated.

How does the brain perform multisensory inference? Researchers have conducted many relevant experiments [91,92] on multimodal inference in complex environments to explore the principles of neural computation. In complex natural environments, the forced fusion of signal sources can be detrimental, and the brain should strive to balance integration and segregation based on the underlying causal structure [93]. Researchers have used Bayesian causal inference methods to localize signal sources for information integration and separation. Many research efforts [94,95] have combined functional magnetic resonance imaging (fMRI) with probabilistic models of cognition to explore brain cognition. They have discovered some important properties of brain-inspired multisensory reasoning.

Rohe et al. [96] observed brain activity using fMRI in their subjects and fitted a causal inference model to the perceptual data. This allowed them to better analyze the mapping between brain activity and the various spatial estimates predicted by the model. They tested brain cognition using different auditory and visual sources and found that at the bottom level (the sensory cortical region), the brain tends to process signals as separate sources. However, at higher levels, the brain integrates sensory signals based on potential common sources. This hierarchical structure reflects the brain’s progressive processing of uncertainty regarding signal sources. Experimental results show that brain-inspired causal inference demonstrates hierarchical characteristics. Some regions predominantly utilize a single perceptual modality, while others exhibit the computational complexity required for causal inference. This suggests that multisensory perception in brain cognition arises from the combined action of multiple processes and networks of interacting regions. Each process relies on different assumptions about the causal structure of the environment and performs distinct computations based on these assumptions [97].

Based on the principle of brain-inspired multisensory perception, causal-based deep learning models can be constructed. Researchers designed a multi-layered neural network structure where the underlying network processes basic attributes and low-level features in the raw input, such as the edges and textures of an image or the waveform of a sound. The deeper network layers are responsible for processing more complex and abstract features. They draw an analogy between brain-inspired multisensory reasoning and DNNs, utilizing this modular design to construct various modules for processing different types of perceptual signals and embedding causal inference modules to integrate information from multiple perceptual signals (multiple data sources or modalities). For instance, Deshpande et al. [98] employed deep structural equation modeling (SEM) to process multimodal data, such as text, images, and graphics. Klaassen et al. [99] proposed a multimodal model for estimating causal effects on unstructured data. Zang et al. [100] constructed a multimodal causal inference framework to decouple causal and confounding features in visual and textual modalities.

Multisensory integration helps the brain detect the source of an event and accelerate the response. Stein et al. [101] suggested that the number of impulses elicited by a single stimulus in a single neuron is significantly different from the number of impulses elicited by a multimodal combination of stimuli. This means that multisensory integration can either enhance or inhibit neuronal responses. Multisensory stimuli compete for the brain’s attention and the propagation pathways that produce behavioral actions when stimulated. This competition positively affects both detection accuracy and response speed. Multisensory neurons are distributed at various levels in the brains of humans and other mammals, with a particular concentration in the superior colliculus (SC). The SC receives signals from multiple multisensory neurons and integrates all the information. Correspondingly, in deep learning models, different weights are assigned to various perceptual signals. Causal inference is used to help understand and determine the importance of these signals, which allows the model to prioritize which signals to process. Important signals are enhanced, while unimportant ones are suppressed. The attention mechanism is employed to simulate the brain’s selective attention, achieving effective information integration and improving decision-making accuracy.

Memory function is also an indispensable part of processing multi-perceptual information. Brady et al. [102] argued that different individual differences lead to different working memory capacities. Therefore, working memory is a fundamental cognitive capacity in the brain, which both shapes and limits our ability to process information across different domains. Lavelle et al. [103] explored working memory for vision and found that hybrid search performance for multiple targets decreases as the number of retrieved targets increases. This indicates that increasing memory capacity reduces retrieval accuracy and lengthens search response time. Drew et al. [104] argued that as the set of target memories grows, the probability of misrecognizing a confounding factor as a target and the probability of missing a target increase accordingly. Saltzmann et al. [105] experimentally tested the effect of behavioral changes on confounder memory in mixed search tasks. Adam et al. [106] argued that working memory and long-term memory are utilized in conjunction in daily life. These studies collectively emphasize the importance of memory in target retrieval and distractor recognition. In the multisensory condition, different perceptual processes are recognized in primary cortices. Matusz et al. [107] suggest that multiple sensory stimuli can significantly enhance memory. For example, consistent semantics from visual information of a cow and auditory information of a “moo” can improve the accuracy of target judgment. Conversely, inconsistent multisensory semantics reduce the accuracy of judgment. In deep learning models, different modal input information affects the model’s judgment, making it crucial to ensure that multimodal information is causally and semantically consistent.

We believe that understanding the multisensory computational processes of brain functioning contributes to a deeper comprehension of the complexity and diversity of human cognitive processes. This, in turn, helps in building sophisticated models to manage real-world multimodal data. Additionally, decoding the brain’s perceptual processes can help us develop more interpretable models.

## Basic of Causal Inference

### Three levels of causality

Causality typically pertains to the interaction between 2 events, where one event is considered the causal and the other the effect. This relationship is objective and independent of human subjective will. “The Book of Why” [108] delves into causal theory and introduces the concept of the ladder of causality, which distinguishes and describes 3 levels of causality:

The first level: Association, which relies on passive observation to identify patterns and explore correlations between variables rather than establishing true causal relationships. In daily life, people often confuse correlation and causality, but strictly speaking, their meanings are completely different. Correlation only reflects the interrelated interaction between 2 events, while causality has a strict chronological order, with the cause preceding and the effect following, leading from the cause to the effect. Confounding factors that influence the relationship between 2 variables are often referred to as confounders.

The second level: Intervention, which involves not only passive observation but also active changes. Interventions aim to change the distribution of data by manipulating variables to minimize the impact of confounding factors and deduce true causality. This level is more concerned with the total or average causal effect of a group rather than individual effects. There are 2 main methods of intervention: randomized controlled trials and observational studies. Randomized controlled trials can face practical challenges. So there is the second intervention called the do-operator. This approach does not require experiments but rather measures the true causal effect from the observed data using the appropriate adjustment formula.

The third level: Counterfactual, which is considered the highest level of the existing causal ladder. Unlike interventions, counterfactuals focus on answering questions about what would have happened if the observed situation had been different. This requires the ability to reason about a hypothetical world.

### The basic paradigm of causal intervention

To ensure accurate causal inferences and eliminate the influence of confounders, researchers have developed various causal inference frameworks [60]. The 2 most commonly utilized approaches are the POM and the SCM.

#### Potential outcomes model

One of the most commonly used causal inference frameworks is the POM [109], also known as the Rubin causal model (RCM), which has applications in fields such as economics, computer science, and biomedicine. Potential outcomes refer to outcomes that are not necessarily observed. When examining a real-world scenario, only one course of action can be taken, leading to a specific outcome. The observed outcome represents the factual, while the alternative outcome that was not chosen is referred to as the counterfactual. To ascertain causal effects, a crucial evaluation metric in causal inference known as the individual treatment effect (ITE) is introduced. ITE helps identify the causal impact stemming from a specific intervention aimed at an individual or a group in causal inference. For an individual denoted as *i*, the formula for independent causal effect is as follows:

$$\tau_{i}=Y_{i}\left(1\right)-Y_{i}\left(0\right)$$ (1)

where *Y*(*t*) represents the potential outcome when the treatment *T* = *t*. *Y*(*0*) represents the outcome for an individual when not receiving the treatment (treatment = 0), and *Y*(*1*) represents the outcome for an individual when receiving the treatment (treatment = 1). As diverse potential outcomes for individuals exist, *Y_i_*(*t*) is regarded as a nonrandom variable. The subscript *i* indicates that attention is focused on a single individual *i*, and the potential outcome *Y_i_*(*t*) is deterministic. A fundamental challenge encountered in computing independent causal effects is the inability to observe both *Y_i_*(1) and *Y_i_*(0). To determine the difference between 2 outcomes, a counterfactual approach must be used. The potential outcome framework, which employs mathematics and computation to elaborate the theory, is distinct from Lewis’ counterfactual framework [110] that uses language to elaborate a theoretical model. This framework presents propositions, hypotheses, and conclusions in causal inference clearly and intuitively, which is more helpful for researchers to learn systematically. However, as a theory with strong mathematical logic, this framework requires certain premise assumptions [60,109].

Assumption 1: SUTVA: Stable unit treatment value assumption (SUTVA) states that each unit is independent and that units do not affect each other. For each unit, different versions or forms of each treatment do not lead to different potential outcomes. This assumption has 2 implications:

Unit independence. The stability of the intervention is emphasized, meaning that the potential outcomes of any unit are not affected by the interventions of other units. For example, if the effects of drug *A* are studied, the outcome of one patient taking drug *A* will not change depending on whether or not other patients are taking drug *A*.

Treatment consistency. There should be no different forms or versions of the intervention that each unit receives that could lead to different potential outcomes. For example, if different doses of drug *A* lead to different outcomes in the clinical trial, then different doses of drug *A* should be treated as different treatments.

Assumption 2: Ignorability assumption: The ignorability assumption, also known as the unconfoundedness assumption, centers on the idea that, given a background variable *X*, the treatment assignment *W* is independent of the potential outcome *Y*. The assumption is based on the following formula. The formula is expressed as:

$$W⊥\left(Y\left(0\right),Y\left(1\right)\right)|X$$ (2)

That is, 2 individuals with the same background should have the same potential outcome regardless of the treatment they actually receive. The converse can also be said of individuals with the same background, that their probability of receiving treatment is the same, independent of their potential outcome. For instance, if 2 patients have the same background variable *X*, the distribution of their potential recovery outcomes (health status with and without treatment) should be the same regardless of whether they receive treatment or not. The probability of receiving treatment should also be equal.

Assumption 3: Positivity assumption: The implication of the regularity assumption is that for any variable *X*, the intervention allocation *W* is not constant. The formula is expressed as:

$$P\left(W=w|X=x\right)>0,\forall w\;\text{and}\;x$$ (3)

For any value of the variable *X*, the intervention allocation *W* will be an uncertain probability greater than zero. This allows every variable to be addressed by each intervention. If we consistently assign a fixed intervention to an observed object *X*, then the outcome of that object *X* under other interventions is unobservable. For example, regardless of a patient’s variable *X*, they will have a certain probability of receiving either drug *A* or drug *B*. There will not be a situation in which only drug *A* and not drug *B* is used for a certain class of people. Overall, the positivity assumption reveals the variability of the intervention. The distribution of the intervention should be variable rather than fixed. This assumption ensures that all possible treatment outcomes can be observed to effectively estimate causal effects.

#### Structural causal modeling

**Fig. 2.**
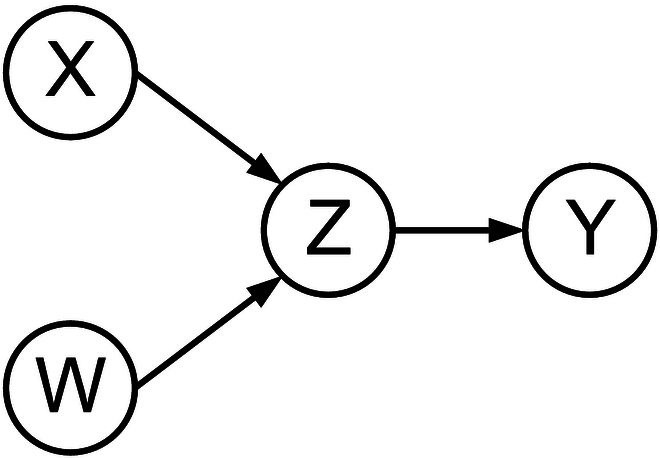
Causal instance graph in structural causal model.

SCM [111] is a methodology that aims to investigate causal relationships by constructing causal graphs and structural equations. The SCM is a ternary model *M* =  < *U*, *V*, *F*>, consisting of 3 primary components: (a) the set of exogenous variables *U*, (b) the set of endogenous variables *V*, and (c) the set of mappings between variables (denoting the set of functions) *F*. External nodes that are not connected to any other node, i.e., nodes without any parent node, are referred to as exogenous variables. For instance, in Fig. 2, *W* and *X* belong to the exogenous nodes category. On the other hand, an internal node with a parent node is known as an endogenous variable, such as *Z* in Fig. 2. Each endogenous variable is a child node of an exogenous variable. The edges in the graph correspond to mapping relationships between variables. To understand SCM, we need to understand 2 important concepts, SEM and graphical causal modeling (often called causal graphs).

Structural equation modeling. The earliest economists and social scientists used SEM to define causality [55]. Haavelmo et al. [112] formally introduced SEM in economics in 1944, emphasizing the importance of incorporating stochasticity in the system into the model. SEM [113,114] was initially developed to depict the relationships between variables in an economic system. It typically involves linear equations with a stochastic disturbance term added to represent unobserved factors or noise. The model is expressed in the following formula:

y=βx+ε (4)

where *β* is the parameter that quantifies the relationship between *x* and *y*. *ε* is the random perturbation term that represents the error present in the system. However, the algebraic equation is a symmetric process. When interpreting this equation, a change in *x* affects *y*, and a change in *y* affects *x* in turn. This implies that we are still unable to correctly express the causality implied in this process [115]. In order to express the directionality of the process, Wright et al. [116] used a graph to visualize the relationship of the variables in the equation. This graphical representation is known as a path diagram. In a path diagram, the connection from cause to effect is represented by directed edges, and the strength of this relationship can also be quantified by path coefficients. Showing causal relationships and paths between variables by constructing intuitive graphical representations helps researchers to build and understand complex causal structures.

After this, Pearl [111] proposed d-separation, a method for graphically discriminating conditional independence between variables. The independence between variables is determined by detecting whether a path in a path graph is blocked by a certain set of nodes. Overall, both path graphs and d-separation are essential tools in SEM. Although some methods have been added to improve SEM, it is still primarily used for linear analysis and is unable to express relationships in nonlinearly dependent systems. In order to overcome this limitation, “effect” needs to be freed from algebraic representation and redefined as an ability to represent the transfer of changes between variables. Pearl [117] proposed an extension to the traditional SEM, constructing a simulation-based intervention to estimate causal effects in nonlinear and nonparametric models. The equations of the extended functional causal model are represented as follows:

$$x_{i}=f_{i}\left(pa_{i},\mu_{i}\right),i=1,\ldots ,n$$ (5)

where *pa_i_* represents the set of variables that directly determine the value of *x_i_*, while *μ_i_* denotes the error or interference caused by omitted factors. Each equation of this form signifies an autonomous mechanism known as a structural model. When each variable in the model has a unique equation that delineates how its value is determined by other variables (which may act as the explained dependent variable), the model is referred to as an SCM [111]. Mathematically, the algebraic equations illustrate the corresponding static relationships between the variables, whereas the structural equations themselves embody an effective causal structure, capable of describing how the system responds to interventions. In general, SEM uses linear or nonlinear models to describe relationships between variables that are not necessarily causal. SCM is a causal extension of SEM that can establish a clear causal relationship for each variable by intervening to set the variable to a specific value and observing the response of the other variables in the system.

Causal graphical model. A causal graphical model, also known as a path diagram or causal BN, is a probabilistic graphical model used to causally encode hypotheses about the data generation process. Essentially, it is a probabilistic graphical model that incorporates causal relationships. Typically, DAGs are employed to construct causal graphical models. In these graphs, each node represents a variable, and each directed edge signifies the direct effect of one variable on another, with this directionality reflecting the causal relationship between variables. To fully comprehend causal graphical models, it is essential first to understand the concepts related to probabilistic graphical models.

Probabilistic graphical models are the basis of causal graphical models, integrating probability and graph theory to represent joint probability distributions between model variables [118]. In this model, nodes represent variables, edges represent dependencies between variables, and the continued products of variables represent the joint probability distribution within the models. This clear representation enables the visualization of the relationship between variables and their structure, eliminating the need to model complex joint probability distributions and reducing the volume and complexity of the model. Probabilistic graphical models are categorized into directed graphs (BNs) and undirected graphs [Markov random fields (MRFs)].

Bayesian networks. BN [119] is a probabilistic graphical model that utilizes DAGs to represent dependencies between variables. It is a DAG network. Nodes represent the variables under consideration, and directed edges represent the influence between variables. The degree of their influence is represented by the conditional probabilities attached to the parent and child nodes with the conditional probability equation:

$$P\left(X_{i}|pa_{i}\right)$$ (6)

where *pa_i_* is the set of parent nodes of variable *X_i_*. The joint probability distribution of the network can be decomposed as:

$$P\left(X_{1},\;X_{2},\ldots ,\;X_{N}\right)=\prod_{i=1}^{N}‍P\left(X_{i}|pa_{i}\right)$$ (7)

The joint probability distribution represents the probability of all possible states of the variables. BNs express the dependencies between events and the likelihood of different events occurring. Unlike the original joint distribution model, BNs compute and store only the conditional probabilities given a parent node, reducing the number of parameters and the complexity of model computation.

Markov random field. An MRF is an undirected graph model where each node represents a variable, and the edges between nodes depict interactions between variables, reflecting dependencies among variables [120]. In an MRF, a subset of nodes with edges connecting any 2 points is called a clique. The joint probability distribution connecting multiple variables can be decomposed into a product of multiple factors based on the cliques. The joint probability distribution is expressed as:

$$P\left(x\right)=\frac{1}{Z}\prod_{q\in C}‍\psi_{q}\left(x_{q}\right)$$ (8)

Here, *q* denotes a clique, and *C* is the set of all cliques in the graph. *ψ_q_*(*x_q_*) is the potential function of the clique *q*, defining the interactions of the variables *x_q_* within the clique. *Z* is the normalization constant, also known as the partition function, which ensures that the sum of the probabilities of all possible states equals one.

Both models have their own advantages in specific scenarios. MRFs emphasize the interrelationships between variables and are suitable for capturing complex dependency structures. BNs emphasize the directionality of variable relationships, describing how one variable is affected by another. Although the DAG structure of BNs forms the basis of SCMs, BNs do not directly represent cause and effect. Instead, BNs express dependencies between variables, calculate statistical correlations, and are used for probabilistic inference, with their algebraic operations being reversible. In reality, however, causality is irreversible, so it constitutes a graph structure that can only reflect conditional independence. In fact, it is computationally equivalent for arbitrarily pointing variables and is unable to distinguish the direction of their correlations or express causality between variables. Nevertheless, the structural properties of their underlying DAG endow them with the potential for causal interpretation. Pearl [55] proposed the concept of causal BNs, which ignore probabilistic information and focus instead on more natural and reliable causal information. In a causal BN, each variable satisfies Markov independence [121], meaning that each variable is conditionally independent of other antecedent variables given the set of parent nodes. That is, each current state is independent of other past states, given its direct previous state. This property enables the effective inference of direct causal relationships between variables. Additionally, causal BNs facilitate causal inference by simulating external interventions through do-calculus and observing their effects on different variables.

#### Difference between POM and SCM

Both frameworks are equally expressive, but they differ in their specific concepts and practical applications. The POM estimates the actual intervention effect by considering potential outcomes under different interventions. On the other hand, the SCM explores the causal relationship between variables by constructing causal graphs. POMs offer the advantage of elaborating causal relationships through symbolic language and probabilistic forms of expression, enabling a more precise expression of causality. However, it cannot provide a mathematical model to derive causal rules, which means that the constraints it proposes may not be complete. So, there is no guarantee of its accuracy. The graphical model language utilized in SCM represents causal relationships more intuitively, allowing for the use of prior knowledge to analyze and explain complex relationships between variables and determine confounders more accurately. Furthermore, SCM assumes that all causal relationships can be represented by a DAG, which by default makes the data conditionally independent. POM, on the other hand, allows modeling and inference of various causal relationships without the restriction of conditional independence. It is also for this reason that the do-operator (“Do-operator” section) in SCM does not apply to POM.

## Standard Paradigms of Causal Intervention

This section describes the standard symbolic representation used for causal intervention, the do-operator, and 2 common standard paradigms for intervention: back-door adjustment and front-door adjustment, which are used for different premises.

### Do-operator

**Fig. 3.**
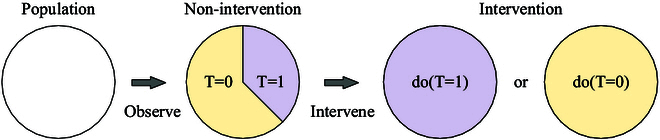
Theoretical schematic of the do-operation.

The do-operator serves as a symbolic representation in causal learning, denoting the intervention on a variable *T* to set it to a specific value *t*, denoted as *do*(*T* = *t*). This operation, commonly referred to as causal intervention, enables the manipulation of variables to explore their causal effects. “Conditioned on *T* = *t*” represents a passive observation, indicating a focus solely on observing changes in the variable when *T* is set to *t*. In contrast, the use of *do*(*T* = *t*) signifies an active alteration in the variables, effectively imposing the value of *T* to be *t*, irrespective of its original state. This distinction is illustrated in Fig. 3. This do-operator facilitates the comprehension of causal effects within the framework of intervention.

### Back-door adjustment

Back-door path: A path between nodes *X* and *Y* that begins with an arrow pointing to *X*, such as *X* ← *Z* → *Y*.

**Fig. 4.**
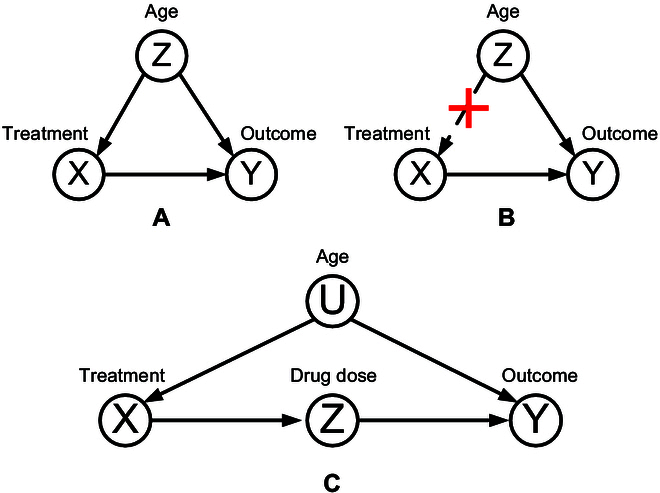
Example of causal graphs. (A) Causal relationship between the treatment, treatment outcome, and the confounder—age. (B) Causal graph after removing the causal path from the age to the treatment. (C) Causal graph after adding the intermediate variable drug dose *Z*.

Back-door criterion: If the set of variables *Z* satisfies the following conditions: (a) it does not contain descendant nodes of *X*, and (b) it blocks every path between *X* and *Y* that contains a path to *X*, then *Z* is said to satisfy the back-door criterion of (*X*, *Y*). A common causal graph containing back-door paths is shown in Fig. 4A. To investigate the true causal relationship between treatment method and treatment outcome, we utilized an intervention to eliminate the causal links of confounders to treatment method *X*. The resulting causal graph is displayed in Fig. 4B.

Back-door adjustment: The purpose of back-door adjustment is to block all other spurious paths between *X* and *Y*, conditional on node *Z*, when determining the causal relationship between *X* and *Y*. This ensures that the directed path from *X* to *Y* is not disturbed. Before providing the formula, it is crucial to understand the meaning of the do-operator. The probability of *Z* = *z* given *Y* = *y*, where *Y* can take on any value, and thus the premise *Y* = *y* is possible, is represented by *P*(*Z* = *z*| *Y* = *y*). Conversely, *P*(*Z* = *z*| *do*(*Y* = *y*)) represents the probability distribution of *Z* = *z* when *Y* has been fixed at *y*. This intervention alters the original data distribution.

To aid in comprehending the formula, we will now introduce 4 rules frequently utilized in deriving formulas [33] for causal inference.

Rule 1: If the variables *W* and *Y* are unrelated, then:

$$P\left(Y|\mathrm{do}\left(X\right),Z,W\right)=P\left(Y|\mathrm{do}\left(X\right),Z\right)$$ (9)

That is, if the variable *W* is statistically independent of the variable *Y*, then any change in *W* does not affect the probability distribution of *Y*. For instance, in the functioning of a smoke alarm, once we determine the state of the mediator *Z* (Smoke), the variable *W* (Fire) becomes independent of *Y* (Alarm).Rule 2: If the variable *Z* blocks all back-door paths between (X, Y), then:

$$P\left(Y|\mathrm{do}\left(X\right),\;Z\right)=P\left(Y|X,\;Z\right)$$ (10)

That is, if *Z* satisfies the back-door criteria from *X* to *Y*, then conditional on *Z*, *do*(*X*) is equivalent to *X*.

Rule 3: If there is no causal path from *X* to *Y*, then:

$$P\left(Y|\mathrm{do}\left(X\right)\right)=P\left(Y\right)$$ (11)

That is, if there is no causal path from *X* to *Y*, we can assume that intervening on *X* does not impact the probability distribution of *Y*.

Rule 4: If there is no confounders between *X* and *Y*, then:

$$P\left(Y|\mathrm{do}\left(X\right)\right)=P\left(Y|X\right)$$ (12)

That is, if there are no confounders among the variables, then the intervention does not change the probability distribution.

The formula for the corresponding back-door adjustment is represented as Eq. 13, where *Z* meets the back-door criterion of (*X*, *Y*). This formula can be utilized to calculate the causal relationship between *X* and *Y*. The specific derivation process of the formulas can be referred to Pearl et al. [124].

$$P\left(Y=y|\mathrm{do}\left(X=x\right)\right)=\sum_{b}‍P\left(Y=y|X=x,Z=z\right)P\left(Z=z\right)$$ (13)

### Front-door adjustment

Front-door path: *A* path between node *X* and node *Y*, starting with the arrow pointed from *X*. For instance, *X* → *Z* → *Y*.

Front-door criterion: Front-door criterion is satisfied when the variable set *Z* meets the following conditions: (a) all paths from *X* to *Y* are blocked; (b) there are no back-door paths from *X* to *Z*; and (c) all back-door paths between *Z* and *Y* are blocked by *X*. If *Z* satisfies these conditions, it is said to meet the front-door criterion for (*X*, *Y*).

Front-door adjustment: We add an intermediate variable drug dose *Z* between treatment *X* and treatment outcome *Y*. The causal graph is shown in Fig. 4C. Suppose that data related to age *U* are not available, and therefore the true causal effect cannot be obtained by blocking the back-door path *X* ← *U* → *Y*. In such a case, the front-gate adjustment method can be employed.

After introducing the basic concepts, we then analyze this causal graph to derive the results of *P*(*Y* = *y*| *do*(*X* = *x*)). As we analyze the front-door path *X* → *Z* → *Y*, we notice that it can be separated into 2 causal paths, *X* → *Z* and *Z* → *Y*. The former is a single causal path with no confounders or back-door paths. The complete path of *Z* → *Y* involves the confounder (*U*, *X*) which satisfies the back-door criterion, where the age *U* is assumed to be unknown.

The formula for the front-door adjustment is Eq. 14.

$$\begin{array}{l} P\left(Y=y|\mathrm{do}\left(X=x\right)\right)\\ \;=\sum_{z}‍P\left(Z=z|X=x\right)\sum_{x^{\prime }}‍P\left(Y=y|X=x^{\prime },Z=z\right)P\left(X=x^{\prime }\right) \end{array}$$ (14)

where *Z* satisfies the front-door criterion of (*X*, *Y*). *x* is distinguished from *x*^′^, which requires traversing all possible values of *x* and summing them, and then multiplying them with the first term of the formula. The specific derivation process of the formulas can be referred to Pearl et al. [122].

### Difference between front-door adjustment and back-door adjustment

To effectively derive the causal relationship between the cause *X* and the effect *Y* using the back-door formula, it is essential to identify the confounding factor *W*. Back-door adjustment relies on this knowledge. In contrast, front-door adjustment involves identifying a specific variable *M* that has a causal relationship between *X* and *Y* when the confounders are unobservable. The causal relationship between *X* and *Y* is derived by calculating the causal relationships between *X* and *M*, and between *M* and *Y*. In the real world, most tasks and datasets are complex, and not all confounders can be observed. Therefore, when facing such tasks, we often resort to using more front-door adjustment formulas.

## Large Model and Causality

In the field of NLP, LLMs offer a capability for addressing causal problems through judgments about logic and the exploration of causal relationships. We posit that LLMs provide a data-driven approach that enables natural language to understand causality and even reason about causal logic. Numerous studies have investigated the causal reasoning abilities of large models. Liu et al. [123] discussed the performance of LLMs and related work on model understanding, commonsense reasoning, counterfactual reasoning, and more. Zhou et al. [68] explored the integration of causal and deep generative modeling, delving deeply into the causal principles within large-scale generative models. Zhou et al. [124] analyzed the primary reasons for inefficiencies in LLMs reasoning and proposed a classification method to optimize efficiency at the data, model, and system levels. In this section, we present recent advances in large models. We discuss whether these models are capable of causal reasoning and summarize the experiments and conclusions of related studies. Finally, we highlight the enhancement of large models in 2 causal subdomains: causal discovery tasks and causal inference tasks.

### Recent advances in large models

Large models refer to machine learning models with a large number of parameters and complex computational structures. These models are typically built on DNNs, containing billions of parameters, and are pretrained on large-scale training data before being fine-tuned for various downstream tasks. The purpose is to train the model with massive data to improve its predictive performance and generalization ability, enabling the model to perform well in diverse and complex environments. Large models, due to their large-scale parameters, training data, and complex network structures, not only exhibit superior predictive performance and generalization but also enable multitask learning and transfer learning through pretraining on large-scale unlabeled data and fine-tuning for various tasks. Large models are now widely used in various fields such as computer vision, NLP, recommendation systems, economic studies, and more.

Existing large models are mainly categorized into natural language-based LLMs [125], vision-based large vision models [126], and multimodal large models [127,128] based on vision-linguistic. LLMs are used in the field of NLP and have been trained on large-scale corpora to grasp various word structures and contextual contexts. In 2018, OpenAI’s LLM GPT-1 [129], showcasing the groundbreaking method of generating high-quality text through pretraining and fine-tuning, marked a pivotal advancement in NLP. Subsequently, in 2020, GPT-3 was unveiled [130], showcasing the remarkable text generation capability of large models and their capacity to handle multiple types of natural language tasks. Based on this, OpenAI released an iterative version, GPT-4 [131], in 2023. This version comprehensively improved the model’s comprehension and generation capabilities and introduced image–text multimodal information processing. With the successive releases of the GPT series, more teams have proposed their LLMs. Google developed Bard [132], a chatbot based on LLMs, and Huawei introduced the Pangu models [133], among others. Large vision models are employed in the field of computer vision to accomplish various visual tasks such as recognition, tracking, and detection. Notable examples include the VIT series [134] developed by Google. Multimodal large models, which can handle multiple types of data such as vision, text, and speech, aim to integrate the capabilities of different information formats to enhance model understanding. Prominent examples include MiniGPT-4 [135], Google’s visual language model PaLM-E [136], and OpenAI’s newly released text-to-video model SORA.

### Can large models perform inference

**Fig. 5.**
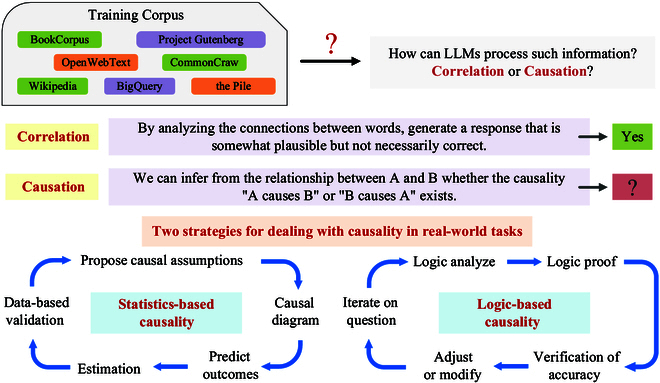
Illustration of the exploration of causality in large language models.

Researchers training large models on extensive corpora have discussed the existence of correlations within the corpus as well as deeper causal relationships [137]. These 2 types of relationships are fundamentally different in their definitions. It is certain that the training process of large models takes word-to-word connections into account, providing relatively reasonable, though not necessarily correct, answers based on these connections. However, whether large models can observe causal relationships between 2 variables and determine their causal direction remains an open question that requires further exploration. Traditional approaches to real-world causal problems can be categorized into 2 types: statistics-based causality and logic-based causality [138]. Statistics-based causality is a data-driven approach that focuses on numerical relationships and correlations between variables. It infers causality by examining the statistical properties of these relationships and usually uses causal graphs to represent them. This approach provides a data-based perspective on causality. In contrast, logic-based causality relies on logical rules to determine causal relationships. It involves logical analysis and reasoning to understand the problem and establish causality. Figure 5 illustrates the process of analyzing and exploring causality using these approaches. In order to explore causality in large models, we have made a compilation and analysis of related studies using causality-based LLMs as an example.

One of the advantages of LLMs today is their user-friendliness, as they do not require unstructured data to be converted into machine-readable language before performing other tasks. Since both the input and output of LLMs consist of text, we can only verify changes in their output by using various text inputs to assess the presence of causal inference in their decision-making process. Zhong et al. [139] and Nori et al. [140] used benchmark tests and question-and-answer evaluations to score large models by evaluating the accuracy of their answers to a set of questions. This approach aimed to analyze the robustness and inference performance of LLMs. However, this method of judgment has certain limitations, as we cannot determine whether the answers provided are based on reasoning or other factors.

To verify the effectiveness of LLMs in identifying causal relationships in data, Hobbhahn et al. [141] provided examples of different types of natural language to test whether LLMs can identify causal relationships in a natural language setting. The experiments found that larger models produce better results. However, introducing interference results in a decline in reasoning performance. This demonstrates the importance of data format for large models, not just the data content. This may suggest that there is still room for improvement in the causal inference abilities of large models. Of course, this study was conducted on the GPT-3 model, and in more advanced GPT-3.5 and GPT-4 models, the reasoning capabilities of LLMs have been further enhanced. Kıcıman et al. [138] experimentally analyzed the performance of LLMs in causal reasoning tasks, employing various testing strategies such as benchmarking and memory testing to evaluate model performance. For real-world natural language problems, LLMs achieved high accuracy in counterfactual reasoning benchmark tests and in identifying necessary and sufficient causes. However, for tasks that rely on understanding human factors, such as assessing the normality of behavior, the LLMs performed poorly. In most contexts, GPT-4 demonstrates the ability to reason, model different scenarios, and answer hypothetical questions based on textual cues. For instance, when asked what would happen if a person caught a water balloon, the LLMs correctly noted that the person might get wet from the balloon breaking. However, when some commonsense information is obscured, GPT-4 produces incorrect answers due to limitations in understanding. For example, when asked what would happen if a person walked on a bed instead of on the street, GPT-4 assumed that the path to the destination had changed from “on the street” to “on the bed”, but that it would still be possible to reach the destination, thus providing the answer “would be late”. This suggests that the lack of commonsense data in the training dataset impacts the construction of causal structures by LLMs. The experiments demonstrate that, regardless of whether current LLMs are fully capable of causal reasoning, they do have a complementary and augmenting effect on aspects of causal tasks that require human reasoning. LLMs can provide significant assistance in understanding causal scenarios and recognizing causal constraints. Zečević et al. [142] argued that LLMs are trained on textual data rather than physical measurement data, making it challenging for them to perform inductive inference from the perspective of data generation, and LLMs do not explicitly construct any causal structural equations. Therefore, they argue that LLMs do not possess causal inference abilities. During empirical analysis, it was found that fine-tuned LLMs perform reasonably well in causal inference tasks. The authors suggest that this is because causal facts already exist in the large-scale training data, which are captured by the large models, enabling them to provide correct answers to some inference questions. However, in more complex problems, true conclusions may still be elusive. Jin et al. [143] developed a new causal large dataset designed to isolate memory effects. This dataset extensively covers all levels of the causal ladder. A ternary of causal queries, graphs, and data containing real answers was constructed in the formal part to ensure the recognizability of the questions. In the natural language part, the causal model and data are linguistically transformed, converting symbolic variables and models into natural language to describe the causal process. Experiments demonstrate that the new dataset can effectively address the problem of data contamination and enhance model performance on unseen data. Additionally, the performance degradation of GPT-4 on anticommonsense data highlights the model’s reliance on memory when handling commonsense tasks.

All of these studies suggest that modern large models have demonstrated some capabilities in cognitive tasks and logical inference. By pretraining and fine-tuning on large amounts of data, they can effectively capture semantic and logical relationships within the data, performing well in tasks such as semantic understanding and question answering. However, there is still room for further improvement in cases that require inference about complex logical relationships and data dependencies. However, this ability relies more on a priori memory from training with large amounts of data and can still be further improved in some cases where complex logical relationships and data dependencies need to be reasoned about.

### Causality in large models

Kıcıman et al. [138] argued that causal tasks in inference systems can be broadly categorized into 2 main types. The first type is causal discovery, which involves determining the causality of the changes in variables. These tasks typically rely on covariates to uncover underlying causal relationships. The second type is causal inference, which focuses on characterizing the strength and structure of known or hypothesized causal relationships. Causal inference relies on logical inference methods to assess causality in the data. In reality, many causal relationships are highly complex, involving numerous hidden variables and unknown mechanisms. This makes modeling causal relationships more challenging, as models may struggle to accurately and comprehensively capture all relevant factors. Large models, trained on massive amounts of data, can learn rich semantic knowledge and variable associations from it. Therefore, large models can be helpful for both traditional causal discovery tasks and causal inference tasks.

Large models with causal discovery: The core of causal discovery tasks is to identify causal relationships among data features. In practical tasks, this is often influenced by data quality issues, making it challenging to identify unobserved data features or determine causal relationships in new domains. Large-scale models possess powerful representation capabilities, enabling them to learn complex data patterns and relationships. Additionally, they exhibit emergence, which allows for a better understanding and analysis of data. By learning high-order statistical features of the data, large models can uncover hidden causal relationships between variables.

The language model has been trained with a wealth of knowledge through a large number of texts. In other words, large models can analyze the metadata associated with variables in the dataset from a causal perspective, providing background information about the variables [138]. Based on these details, they can infer the correct causal structure. Therefore, relying on large models can lead to further identification, interpretation, and recommendations based on the preliminary causal graph obtained from causal discovery, resulting in more accurate causality.

Choi et al. [144] developed the LMPriors framework using metadata generated from language models (e.g., variable names and attribute descriptions). By utilizing the output of the language model as prior information for learning, the framework introduces task-related inductive biases to improve the learning process. The article investigates the application of LMPriors in causal discovery, aiming to determine the direction of causality between 2 variables. Hypotheses are formulated using metadata, which are then integrated into the causal algorithm as additional prior information based on the prior probabilities derived from the language model. This integration is used to compute the posterior probabilities of the final causal direction. Lyu et al. [145] proposed a new paradigm based on LLMs to model the robustness test of causal direction using zero-shot cues. This approach transforms the *P*(*Y*| *X*) and *P*(*X*| *Y*) models into natural language cues describing causal relationships. The experiment successfully identified causal direction in sentiment categorization, providing a new approach to causal inference. Ashwani et al. [146] proposed a new research framework that combines explicit and implicit causal reasoning. Explicit knowledge integration from ConceptNet is used to assist LLMs in comprehending the causality of a scene. Implicit inference patterns from models like BETR are used to offer comprehensive contexts and counterfactuals to the LLMs, assisting in data-driven reasoning. This framework enhances the performance of large models in areas such as causality identification, causal discovery, causal interpretation, and counterfactual reasoning. Vashishtha et al. [147] utilized large models to simulate domain expert capabilities in order to derive the causal order of variables from cue information and context. They proposed 2 causal discovery algorithms: one that orients the undirected edges output by a constraint-based algorithm using the causal order from the LLM, and another that employs the causal order from the LLM as a score-based algorithm.

Large models with causal inference: The core task of causal inference is to infer the magnitude of the effect of one variable on another variable using a known causal structure. Large-scale models possess powerful pattern recognition and data analysis capabilities, allowing them to learn complex patterns from massive datasets. Consequently, they can effectively identify the mutual effects between variables and infer causal relationships between variables. Moreover, due to their large-scale training databases, large models have strong generalization abilities, making them more suitable for various tasks across different domains.

Zhou et al. [68] conducted a causality study for generative LLMs. They argued that LLMs can initially be used as preprocessing tools to analyze observational data and identify potential causal relationships. Furthermore, LLMs can be integrated into the causal analysis process to construct data-driven causal algorithms and enhance the output quality of these algorithms. Tang et al. [148] proposed a multi-agent collaborative framework in which multiple agents play the roles of reasoner agents and evaluator agents. The reasoner agent is responsible for reasoning about a particular problem in a causal manner, while the evaluator agent examines the causal consistency of the solution from noncausal and counterfactual perspectives, as well as the soundness of the knowledge reasoning. This framework aims to improve the accuracy and reasonableness of knowledge reasoning by leveraging an LLM with contextual learning capabilities to solve reasoning problems from the perspective of causal consistency. Chen et al. [149] utilized large-scale language modeling to generate high-quality counterfactual data to enhance the causal representation of the model. Through contextual learning, a wide variety of counterfactual perturbations are generated, and these generated messages are filtered using a specialized teacher model to retain new data that can significantly alter the original labels. This approach results in improved counterfactual robustness in natural language inference (NLI). Feder et al. [150] similarly modeled interventions for spurious features through LLM-based counterfactual augmentation to reduce the model’s dependence on features and thus train more robust text classifiers.

## Application of Causal Discovery

Inferring causal relationships from observational data is recognized as causal discovery, a method that has garnered significant attention across diverse domains, including machine learning, statistical learning, and beyond. Over time, as vast quantities of data have accumulated, the task of causal discovery has encountered increasingly formidable challenges. Data often experience distributional shifts due to differences in sampling conditions or feature representations across different datasets, or from factors such as temporal fluctuations. These conditions often make causal discovery tasks particularly challenging.

In order to effectively identify the causal structural framework of observational data, Huang et al. [151] proposed a principled framework called CD-NOD (constraint-based causal discovery from heterogeneous/nonstationary data). The invariance of causal conditional distribution is determined by a nonparametric conditional independence test, and a causal direction determination algorithm is constructed based on causal independence. The dependencies between causal modules are measured using the Hilbert Schmidt independence criterion (HSIC) to infer the causal architecture and the causal direction between the variables, and efficiently estimate the nonsmoothness driving the causal mechanism. The traditional approach to discovering causal structure typically involves encoding conditionally independent relationships with identical distributions observed in the data. However, this method is limited to identifying overarching causal structures and may overlook more detailed and intricate causal relationships. Guo et al. [152] proposed that a more comprehensive causal structure can be achieved by learning from datasets originating from diverse environments. This article introduces causal de Finetti theorems, which describe the concept of independent causal mechanisms and establish connections between exchangeable data and multi-environmental datasets. This ensures that accurate causal graphs can be derived given a sufficiently large amount of exchangeable data. Li et al. [153] delves into the identification of confounding factors within potential outcomes and aims to mitigate their effects. Causal minimality is presupposed to ensure that the impact of each parent node remains nonzero. Subsequently, a nonlinear structural equation incorporating additive confounders and noise is formulated. This equation is constructed using a nonparametric fitting method, with deconfounding adjustments applied to mitigate confounding effects. Furthermore, an ordering process is integrated to estimate the causal sequence of variables. The model’s versatility is enhanced by incorporating a feed-forward network. To enable efficient posterior inference on observed data, Annadani et al. [154] introduced a scalable Bayesian causal discovery framework that combines stochastic gradient Markov chain Monte Carlo (SG-MCMC) with variational inference (VI). This framework eliminates the need for regularization and facilitates the direct sampling of DAGs from the posterior for both linear and nonlinear models. Determining the causal structure within an environment typically requires expensive real-world interventions. To mitigate the necessity for numerous interventions while estimating a causal model, Sauter et al. [155] introduced a meta-reinforcement learning framework. This framework trains a causal discovery algorithm called meta-causal discovery (MCD), which selects interventions using “listening” actions to ensure their effectiveness. Additionally, it employs structural actions to uphold the current causal structure by imposing specific constraints on intervention actions.

## Deep Learning Algorithms with Causal Inference

**Fig. 6.**
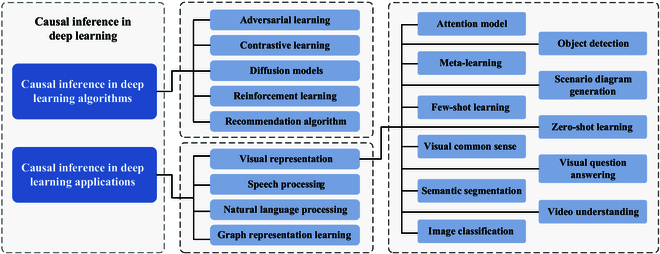
An overview of artificial intelligence tasks based on causal inference.

Achieving the inference capability of AI is an extremely complex problem. In the past, the success of deep learning relied on massive IID datasets and high-performance computing systems. The principle of training on these IID datasets is to identify correlations within the data. However, models trained based on correlations can be unstable, and even small changes to the dataset can cause the model to produce inaccurate results. Therefore, researchers have introduced the concept of causality [61,156] in the hope of enhancing the stability and invariance of deep learning models, enabling them to achieve better generalization ability. Causal learning can establish strong causal links within complex and disordered data, thereby improving the interpretability, generalization ability, and robustness of deep learning algorithms. To illustrate the application of causal-and-effect combined deep learning algorithms, we have divided several different tasks. The overview diagram is shown in Fig. 6. We introduce classical deep learning algorithms that incorporate causal inference, including reinforcement learning, diffusion models, adversarial learning, contrastive learning, and recommendation algorithms. We then discuss the applications of causal inference in specific deep learning paradigms such as speech processing, NLP, graph representation learning, and visual representation tasks. Specifically, for visual representation tasks, we detail 11 distinct categories to provide a comprehensive overview. In this section, we introduce deep learning algorithms that incorporate causal inference, and list them in Table 1.

**Table 1.** An overview of causal inference in classical deep learning algorithms

Model	Publication	Key characteristics	Deep algorithm	Causal method
CADE [162]	AAAI 2024	Adversarial attack, counterfactual, adversarial robustness	Adversarial Attack	Counterfactual
- [161]	MIR 2022	Adversarial sample, adversarial defense, causal inference, interpretable machine learning, transformers	Adversarial attack	Counterfactual
CausalAdv [163]	2021	Adversarial attack, adversarial defense, causal intervention	Adversarial defense	Causal intervention
CausalGAN [166]	ICLR 2018	Generative adversarial network, intervention, image generation	Generative adversarial network	Causal intervention
CAN [167]	2020	Generative adversarial network, causal adversarial network, image generation	Generative adversarial network	Causal discovery
CGNN [168]	2018	Generative neural networks, causal structure discovery, cause-effect pair problem, functional causal models, structural equation models	Generative adversarial network	Causal discovery
Causal-TGAN [169]	2021	Generative adversarial network, tabular data generation	Generative adversarial network	Causal discovery
SCIGAN [170]	NIPS 2020	Counterfactual inference, generative adversarial networks	Generative adversarial network	Counterfactual
*C*^2^*L* [171]	AAAI 2022	Counterfactual, robustness, robust text classification, contrastive learning	Contrastive learning	Counterfactual
PPI [172]	2021	Visual explanations, contrastive learning, model interpretability	Contrastive learning	Causal intervention
GCIL [173]	2024	Graph contrastive learning, causal intervention	Contrastive learning	Causal intervention
CausalDiffAE [178]	2024	Diffusion probabilistic models, counterfactual generation, causal diffusion autoencoders	Diffusion models	Causal intervention/counterfactual
Diff-SCM [179]	2022	Generative energy-based models, diffusion causal models	Diffusion models	Causal intervention/counterfactual
BDCM [180]	SSCI 2023	Diffusion-based causal model, back-door adjustment	Diffusion models	Causal intervention
- [181]	OJSP 2024	Causal processing, diffusion models, generalized speech enhancement	Diffusion models	Causal processing
- [182]	2021	Observational data, interventional data, causal reinforcement learning	Reinforcement learning	Causal intervention
DOVI [183]	NeurIPS 2021	Confounded observational data, causal reinforcement learning	Reinforcement learning	Causal intervention
- [184]	ACC 2023	Gene regulatory networks, reinforcement learning, causal inference	Reinforcement learning	Causal intervention
Deep- Deconf [189]	ACM 2022	Recommender systems, causal inference, multi-cause confounders	Recommendation algorithm	Causal inference
CountER [186]	ACM 2021	Explainable recommendation, counterfactual explanation, counterfactual reasoning, machine learning, explainable AI	Recommendation algorithm	Counterfactual
CEF [187]	ACM 2022	Explainable fairness, recommender systems, explainable recommendation, fairness in AI, counterfactual reasoning	Recommendation algorithm	Counterfactual
Causer [188]	2022	Sequential recommendation, causal behavior discovery	Recommendation algorithm	Causal discovery

### Causal inference-based adversarial learning

Adversarial learning is a research paradigm aimed at maintaining model stability and robustness in the presence of malicious environments or human interference. By adding perturbations to real samples to create new adversarial samples, neural networks can be misled in their judgments. Thus, improving the adversarial robustness of neural networks and enhancing the model’s ability to recognize adversarial samples becomes crucial. Adversarial learning is usually divided into adversarial attacks [157,158] and adversarial defenses [159,160].

Adversarial attack: Adversarial attacks aim to generate adversarial samples more efficiently, typically by manipulating the input samples and thereby inadvertently disrupting the underlying IID data structure. Testing for conditional independence in a complex and multidimensional dataset can be challenging, and detecting conditional independence can be even more difficult. While humans may not perceive these changes acutely after breaking the IID distribution, the modified samples can cause intervention for the model. Causality can mitigate the impact of adversarial attacks on deep learning models. Ren et al. [161] employed a self-attention transformer as a tool to construct a causal model that explains the generation and performance of adversarial samples and proposed a simple and effective strategy to defend against adversarial attacks. Cai et al. [162] proposed a novel adversarial learning framework based on the causal generation method introduced by Pearl [55]. This framework generates counterfactual adversarial examples by altering the distribution through intervening variables while keeping the objective unchanged, addressing the vulnerability of DNNs to well-crafted adversarial examples.

Adversarial defense: Adversarial defense aims to offer more effective protection against adversarial samples. Zhang et al. [163] argued that the root cause of adversarial vulnerability in DNNs is the model’s reliance on false correlations. The authors construct causal graphs to model the generation process of adversarial samples, providing a causal perspective on adversarial vulnerabilities. They propose an adversarial distribution alignment method to formalize the intuition behind adversarial attacks. By eliminating the differences between natural and adversarial distributions, the adversarial robustness of the model is improved.

Generative adversarial network: Building on the foundation of adversarial learning, researchers have proposed generative adversarial networks (GANs) [164,165]. GANs consist of 2 components: a generator, which creates adversarial samples, and a discriminator, which determines whether the input data are real or generated. Kocaoglu et al. [166] proposed a 2-stage causal generative model called CausalGAN, which first trains a causal implicit generative model on binary labels, and then introduces a new conditional GAN to help the generator sample from the correct intervention distribution. Moraffah et al. [167] argued that the causal graph constructed in CausalGAN relies on known labels, making it challenging to apply the model to real-world tasks and difficult to scale with large amounts of labeled data. To address these issues, they proposed a scalable generative causal adversarial network (CAN). CAN is structured into 2 parts: the label generating network (LGN), which learns causal relationships from data and generates samples, and the conditional image generating network (CIGN), which receives labels and generates the corresponding images. Goudet et al. [168] introduced a framework called causal generative neural networks (CGNNs) to learn data distributions with causal construction generators. Wen et al. [169] proposed a data generation architecture called Causal-TGAN, which aims to solve the causal problem in tabular data generation and generate datasets with different variable types. Multiple causal processes are captured by building an SCM, which improves the accuracy of the target data distribution. Bica et al. [170] proposed a hierarchical discriminator called SCIGAN, for estimating counterfactual outcomes at successive interventions. The key idea is to generate counterfactual outcomes through a modified GAN model and learn an inferential model using a standard supervised approach to estimate counterfactuals for new samples.

### Causal inference-based contrastive learning

Contrastive learning is an unsupervised learning paradigm that aims to differentiate data in the feature space at an abstract semantic level. It encodes data of the same class as similarly as possible while ensuring that the encodings of data from different classes are maximally distinct. The core idea is to distinguish samples by their labels or attributes and partition them into different representation spaces, with the distance between them reflecting the degree of similarity. Nowadays, contrastive learning methods are frequently applied in image feature learning, text representation learning, and other domains. Common paradigms include supervised contrastive learning, which uses the actual labels of data samples to generate contrast samples, and self-supervised contrastive learning, which generates contrast samples based on the intrinsic attributes of the data.

Supervised contrastive learning: Choi et al. [171] proposed a causal-based contrastive learning approach to improve the robustness of text categorization models. For unbiased causal identification, candidate tokens are selected based on attribution scores. The causality of these candidate tokens is then verified by evaluating their individualized treatment effect (ITE). To enhance robustness, a multiple reconstruction approach is employed for validation, which involves synthesizing counterfactual samples and making decisions based on the distribution of predictive labels for these samples. This method effectively reduces the reliance on spurious features in deep learning models. Wang et al. [172] applied contrastive learning to visual problems and proposed a causal intervention-based contrastive learning mechanism called proactive pseudo-intervention (PPI). In this approach, pseudo-interventions are synthesized from observational data using contrastive representation learning. This reduces the model’s dependence on image features that are strongly correlated with the target label but not causally related, through proactive intervention. This method addresses the issue of DNNs over-relying on noncausal visual information in image classification while also improving the model’s interpretability and generalization ability.

Self-supervised contrastive learning: Mo et al. [173] analyzed the process of graph generation based on the SCM and found that traditional graph contrastive learning is affected by noncausal information, hindering the learning of invariant representations. To address this issue, they proposed a new graph contrastive learning method called graph contrastive invariant learning (GCIL). The model uses an SCM to describe the graph generation process. The original graph *G* is divided into a set of causal variables *C* and a set of noncausal variables *S*. It is desired that the causal variables remain consistent, while the noncausal variables vary for each enhancement process of the augmented graph. That is, *C_A_* = *C_B_* = *C*, and *S_A_* ≠ *S_B_*. However, current stochastic enhancement strategies usually fail to distinguish between *C* and *S*, which can lead to different predictions for graphs containing the same causal information. The authors thus intervene causally on the noncausal variable *S* to ensure that the variable satisfies the following equation:

$$P^{\mathrm{do}\left(S=s_{i}\right)}\left(Y|C\right)=P^{\mathrm{do}\left(S=s_{j}\right)}\left(Y|C\right)$$ (15)

This intervention method allows for a different noncausal variable *S* that does not affect the predicted outcome *Y* while the causal variable *C* remains constant. Overall, this approach generates causal views to model interventions on noncausal factors from a graph perspective and designs invariant objectives to ensure that these views maintain the same mean and variance in each dimension. It better captures causal information and improves the robustness of the learned representations.

### Causal inference-based diffusion models

The diffusion model, first proposed by Sohl-Dickstein et al. [174] in 2015, systematically and gradually breaks down the structure of data distribution through an iterative forward diffusion process. It then restores the data’s structure using a backward diffusion process to remove Gaussian noise applied continuously to the training images. In 2020, Ho et al. [175] introduced denoising diffusion probabilistic models (DDPMs), which applied the concept of diffusion modeling to image generation for the first time, enhancing the practicality of this learning approach. Standard diffusion models usually have 2 main processes: forward diffusion and reverse diffusion. In the forward diffusion phase, noise is slowly and iteratively added to the image until it becomes completely random noise. The purpose of this process is to map the image to a different space, transforming the complex and unknown distribution of the training set images into a distribution that is well-understood and already sampled. In the reverse diffusion phase, the image is recovered from the Gaussian noise by slowly and iteratively removing the predicted noise at each time step using a series of Markov chains.

Nowadays, the number of studies based on diffusion learning has surged dramatically, covering a wide range of research areas. Poole et al. [176] utilized a pretrained text-to-image 2-dimensional (2D) diffusion model for text-to-3D image synthesis. This approach circumvents the need for 3D training data or modifications to the image diffusion model, thereby addressing the scarcity of 3D data and models. It also underscores the efficacy of the image diffusion model. Yue et al. [177] discovered that the time step of the diffusion model can isolate fine-grained category features. Building upon this finding, they introduced the time-step few-shot learner. By training class-specific low-rank adapters to compensate for features lost during the diffusion process, the model’s performance in fine-grained classification tasks is notably enhanced.

Researchers have integrated diffusion learning with causal innference to tackle the challenges associated with diffusion learning, aiming to enhance model interpretability and meet the requirements for high-quality generation. Enhancing the quality of generated images through diffusion models has been extensively explored. However, the realm of controlled counterfactual generation using diffusion probabilistic models (DPMs) remains relatively uncharted. Komanduri et al. [178] introduced a causal representation learning framework based on DPMs for counterfactual image generation. Meaningful causal variables are extracted from high-dimensional data using a learnable stochastic coder, and the random variables are modeled using reverse diffusion to ensure the decoupling of causal variables. Due to limited labeled data, the authors propose an extensible weakly supervised approach to reduce the need for labeled samples for joint training of labeled and unlabeled diffusion models. Sanchez and Tsaftaris [179] proposed a deep SCM called Diff-SCM for counterfactual estimation from observed image data with a known causal structure. The authors consider the diffusion process as a form of causal weakening. In the forward diffusion process, the endogenous variables gradually transition from their original joint distribution to a fully independent Gaussian distribution. The reverse diffusion process restores the causality between endogenous variables through iterative updates. By inferring latent variables based on the properties of the diffusion model, this approach helps quantify the causal effects of interventions in high-dimensional data. Shimizu [180] proposed a diffusion-based causal inference method that extends the diffusion-based causal model (DCM) by introducing a back-door criterion, enabling accurate sampling from the target distribution even in the presence of unmeasured confounders. Richter et al. [181] used causal diffusion modeling in a speech generation task by treating the diffusion model as an iteratively denoising task in which a DNN is trained to remove progressively added Gaussian noise. A task-adaptive diffusion process that conditions the fractional model on the corrupted input signal is used to address the speech distortion problem in the speech generation task.

### Causal inference-based reinforcement learning

Reinforcement learning typically necessitates 2 prerequisites for achieving optimal outcomes: a substantial amount of adequate data and a well-defined scenario. However, several fields, such as medicine, cannot satisfy these requirements due to a scarcity of data for specific cases, rendering reinforcement learning unsuitable for training. To address these challenges, researchers have introduced the concept of causal reinforcement learning. It is a method of causal inference that can establish causal relationships between data stimulus signals and outcomes. This enables computers to learn relevant information from the data more efficiently. Gasse et al. [182] introduced a causality-based approach that combines offline and online data to enhance model performance. The decision problem in a partially observable Markov decision process (POMDP) is transformed into a causal query problem, where the causal effect of actions on future rewards is inferred through intervention operations to optimize the decision. Wang et al. [183] defined the causal relationships between states, actions, rewards, and confounders using SCM. By introducing confounders into the Markov decision-making process, definitions of value functions and action value functions were established to improve the estimation and optimization of strategies. The de-confounding optimistic value iteration (DOVI) algorithm was developed to reduce the effect of confounders in both scenarios and accurately extract information from confounders when they satisfy the back-door assumption or the front-door assumption observations. In the field of medicine, Alali and Imani [184] proposed a reinforcement learning-based data acquisition strategy aimed at reducing the uncertainty in networks caused by unknown interactions between genes, which helps achieve accurate causal inference of regulatory interactions. A Bayesian inference-based approach is used to infer unknown interactions in gene regulatory networks (GRNs). The gene expression profiles are altered by introducing perturbations to obtain data that can reveal the true regulatory interactions. Optimal perturbation sequences are selected by constructing prior probabilities and maximum a posteriori (MAP) estimates to improve the confidence of MAP inference.

### Causal inference-based recommendation algorithm

Recommendation algorithms are essential for personalized services on the internet and are widely used in e-commerce platforms, social networks, and other systems to suggest products that match users’ preferences [185]. Traditional recommendation algorithms rely on learning correlations in data, which can lead to misleading results. In observational studies, unobserved confounders can introduce systematic bias. To address these issues, causal inference has been introduced in recommendation algorithms. Tan et al. [186] introduced the counterfactual explainable recommendation (CountER) algorithm based on causality, which can identify a less complex and more efficient recommendation explanation through counterfactual analysis. Ge et al. [187] conducted research on the fairness issue in recommender systems using a counterfactual reasoning framework and identified the key features that have a significant impact on fairness. Wang et al. [188] presented a causality-enhanced sequential recommendation framework that incorporates a causal discovery module in sequential recommendation. They constructed a causal graph and trained the causal graph and sequential recommendation model by fitting behavioral data. Zhu et al. [189] model the recommendation task as a multi-cause, multi-consequence (MCMO) inference problem. They consider item exposure and user rating as multi-cause processes and latent outcomes, respectively, approximating the intractable a posteriori probability distributions through VI. Confounding bias is eliminated by estimating user-specific latent variables to control for confounding factors during causal inference.

## Deep Learning Applications with Causal Inference

In this chapter, we explore the applications of causal inference across various deep learning modalities, specifically focusing on downstream tasks for 4 data types: voice (speech processing), text (NLP), graphics (graph representation learning), and images (visual representation). In particular, we divide visual representations into 11 specific tasks such as semantic segmentation, target detection, scene graph generation (SGG), video understanding, etc., and present them in detail. The applications are listed in Table 2. By presenting task-specific applications, we aim to promote research in the interdisciplinary field that lies at the intersection of multiple disciplines and causal learning, providing readers with a clearer understanding of the significance of causal learning applications.

**Table 2.** A partial overview of causal inference in deep learning applications (without visual representation)

Model	Publication	Key characteristics	Deep learning task	Causal method
CFIE [192]	EMNLP 2021	Information extraction, structural causal model	NLP	Counterfactual
- [196]	EMNLP 2020	Named entity recognition, structural causal model	NLP	Counterfactual
CGI [194]	NAACL 2021	Unstructured data, legal text analysis	NLP	Causal inference
CausalBERT [195]	IJCAI 2020	Commonsense generation , causal knowledge graphs	NLP	Causal inference
- [70]	ACL 2022	Causal inference, explainable predictions	NLP	Causal inference
DIVA [193]	2023	Causal effects, latent variable, causal inference	NLP	Causal estimation
CRN [191]	2020	Speech enhancement, speech denoising, neural networks, raw waveform	Speech processing	Causal inference
CISE [190]	2022	Observational inference, deep causal inference, speech enhancement	Speech processing	Causal intervention
DIR [204]	ICLR 2021	Graph neural networks, inherent interpretability, invariant learning	Graph representation learning	Causal intervention
GOOD [205]	2022	Invariance principle, out-of-distribution, structural causal models	Graph representation learning	Causal inference
RCGRL [206]	AAAI 2023	Graph neural networks, graph causality learning, confounding effects	Graph representation learning	Causality learning

### Causal inference-based speech processing

Neural network-based speech enhancement (SE) has shown promising results in improving speech processing and intelligibility. However, traditional SE methods are susceptible to noise interference, leading to increased uncertainty in model predictions. Hsieh et al. [190] modeled the presence of noise as an intervention, using the potential outcome framework to distinguish between clean speech frames and noisy frames, and fed them into 2 mask-based enhancement modules to perform the SE task under noisy conditions. Defossez et al. [191] proposed a causal SE model based on an encoder–decoder architecture optimized in the time and frequency dimensions. They use skip connections to help the encoder–decoder retain more detailed information when processing signal transitions. A causal model based on a unidirectional long short-term memory (LSTM) network is proposed to ensure that only current and previous input data are used for training, thus satisfying the need for causal inference.

### Causal inference-based NLP

NLP is a crucial area of research in deep learning. Feder et al. [70] delved into the significance of causal inference in NLP, highlighting its potential for enhancing model performance, improving robustness, and enabling interpretability. Nan et al. [192] introduced the concept of counterfactual information extraction (CFIE), which enables the identification of deep causal relationships in datasets from a causal perspective. Their approach involves building a unified SCM for multiple information extraction tasks and generating counterfactual samples using the corresponding linguistic structures to calculate more intuitive causal effects. Zhou and He [193] proposed the disentangling interaction of variables (DIVA) framework to reveal the interactions between different variables. It extracted contextual representations from pretrained language models, used a variational autoencoder to determine the posterior distribution of various latent variables, and then employed a variable disentanglement module to disentangle the variables. This process ensures that each covariate only influences its respective target, thereby promoting independence between variables. Liu et al. [194] proposed a graph-based causal inference framework (CGI) that constructs causal graphs based on facts to capture the causal relationships between different variables. Meanwhile, Li et al. [195] developed a large corpus of causal sentences (CausalBank) and proposed a conditional text generation framework based on a collection of causal sentences and a large lexical causal indicator graph. Zeng et al. [196] introduced a weakly supervised approach that divides the sentence into entity and contextual parts from a causal perspective. They generate counterfactual instances by intervening in existing ones.

### Causal inference-based graph representation learning

Graph representation learning aims to represent data as graphical structures, leveraging the topology of the graph and the relationships between nodes to learn feature representations. This approach is frequently used in search, recommendation, and other systems where scene data are used to construct a structured graph. In this context, data are represented as nodes, and the relationships between data points are depicted as edges connecting these nodes. By learning vector representations of nodes, the model can better understand the relationships and attributes within the graph. Graph embedding method [197] is an important method for learning graph representation using deep learning. It can be classified into traditional graph embedding methods that rely on static graphs [198,199] and dynamic graphs [200,201], and methods based on graph neural networks (GNN) [202,203].

Wu et al. [204] addressed the interpretability problem of graph neural networks (GNNs) from a causal perspective to tackle data bias issues stemming from insufficient exploration of causal relationships in GNNs. They proposed discovering invariant rationale (DIR), a method to intervene in training distributions and identify causal explanations that remain consistent across different distributions, thereby filtering out unstable, spurious patterns. Specifically, they developed an explanation generator to partition the input graph into causal and noncausal subgraphs, and a distribution intervener to causally intervene on the noncausal representations. This approach allows the model to infer the invariant causal components effectively. The formula is defined as follows:

$$\begin{array}{c} \min R_{\mathrm{DIR}}=E_{s}\left[R\left(h\left(G\right),Y|\mathrm{do}\left(S=s\right)\right)\right]\\ \;+\lambda \mathrm{Va}r_{s}\left(\left\{R\left(h\left(G\right),Y|\mathrm{do}\left(S=s\right)\right)\right\}\right) \end{array}$$ (16)

The s-interventional distribution is generated by the intervention operation *do*(*S* = *s*), which fixes *S* to a specific value *s*. The s-interventional distribution represents the distribution resulting from the s-interventions. $R\left(\cdot \right)$ calculates the risk under the s-interventional (s-intervention) distribution, and Var*_s_*(·) calculates the risk variance for different s-intervention distributions. By minimizing the risk and variance of risk under different intervention distributions, stable explanatory variables are identified, enhancing the model’s robustness in various data environments.

Chen et al. [205] proposed a novel framework called graph out-of-distribution generalization (GOOD), which addresses this challenge by building SCMs to characterize the graph generation process and identify underlying invariant subgraphs for prediction. Specifically, GOOD decomposes a GNN model into 2 sub-processes, identifying invariant subgraphs and classification. This decomposition employs a contrastive strategy to ensure the recognizability of the invariance principle. Experiments demonstrate that this method achieves state-of-the-art performance in out-of-distribution (OOD) generalization. Gao et al. [206] identified confounding factors in graph representation learning that hinder the model’s ability to learn semantic information. To address this, they proposed a method called robust causal graph representation learning (RCGRL), which aims to learn robust graph representations resilient to confounding effects. RCGRL eliminates confounders by generating instrumental variables and applying an approach based on unconditional moment restrictions to capture discriminative information related to downstream causal predictions. This method effectively addresses confounding effects in graph representation learning, thereby enhancing the performance and generalization of graph representations.

### Causal inference in visual representation

Causal inference is based on the directed relationship between variables and uses interventions, counterfactuals, and other methods to explore the causal relationships among multiple variables. This helps construct more stable and credible model mechanisms, which are widely used in various visual representation domains, such as visual interpretation, scene map generation, image processing, visual question answering (VQA), and other related areas. We discuss each of these areas in detail below and list them in Table 3.

**Table 3.** An overview of causal inference in visual representation learning

Model/module	Publication	Key characteristics	Deep learning task	Causal method
CaaM [207]	ICCV 2021	Causal attention module, visual recognition	Attention learning	Back-door adjustment
- [210]	ICLR 2019	Meta-learn causal structures, representation learning	Meta-learn	Causal intervention
ICIN [211]	2019	Goal-conditioned policy, visual observation, causal induction	Meta-learn	Causal inference
IFSL [216]	NeurIPS 2020	Meta-learn, few-shot learning, causal intervention	Meta-learn	Back-door adjustment
MLM [308]	ICCV 2021	Object detection, automatic drive, masked language models	Object detection	Causal intervention
CIM [238]	ICTAI 2021	Object detection, visual context, causal intervention	Object detection	Back-door adjustment
- [239]	TPAMl 2022	Domain adaptation model, object detection, causal intervention, representation learning	Object detection	Back-door adjustment
MAD [240]	CVPR 2023	Domain adversarial learning, domain shift, causal factors, non-causal factors	Object detection	Causal learning
D&R [217]	AAAI 2023	Few-shot learning, knowledge distillation, few-shot object detection, structural causal model, causal intervention	Object detection	Back-door adjustment
DCFD [241]	AAAI 2022	Unsupervised salient object detection, debiasing framework, causal intervention	Object detection	Back-door adjustment
CMAT [241]	ICCV 2019	Scene graph generation, counterfactual critic, multi-agent policy	Scene graph generation	Counterfactual
TDE [247]	CVPR 2020	Scene graph generation, unbiased learning, counterfactual causality	Scene graph generation	Counterfactual
TsCM [248]	TPAMI 2023	Scene graph generation, causal inference, counterfactuals, representation learning, long-tailed distribution	Scene graph generation	Causal intervention
Causal-SETR [223]	ACCV 2022	Causal intervention, vision transformer, semantic segmentation	Semantic segmentation	Back-door adjustment
CauSSL [225]	ICCV 2023	Semisupervised learning, medical image analysis, image segmentation, causal diagram	Semantic segmentation	Causal diagram
CAUSE [229]	2023	Unsupervised semantic segmentation, causal intervention, self-supervised learning	Semantic segmentation	Front-door adjustment
CausalCellSegmenter [224]	2024	Causal inference, feature aggregation, cell nucleus segmentation, pathology image	Semantic segmentation	Causal inference
CityCAN [208]	2024	Spatiotemporal data mining, causal intervention, attention	Semantic segmentation	Causal intervention
IVG [251]	CVPR 2021	Video grounding , contrastive learning, causal intervention	Video analysis	Back-door adjustment
Causalainer [252]	CVPR 2023	Video summarization, explainability, causal semantics extractor	Video analysis	Causal learning
- [249]	CVPR 2019	Multimodal explanations, video understanding, counterfactual explanations	Video analysis	Counterfactual
TS-PCA [250]	CVPR 2021	Weakly supervised temporal action localization, video understanding	Video analysis	Causal intervention
MCR [100]	CVPR 2023	Video question answering, causal intervention, multimodal causal inference	Video question answering	Back-door adjustment
CaVIR [259]	ICCV 2023	Video question answering, multiple contexts, context-aware	Video question answering	Causal inference
VCSR [265]	ACM 2023	Video question answering, causal inference, cross-modal	Video question answering	Front-door adjustment
LLCP [266]	ICLR 2023	Video question answering, latent causal processes, self-supervised model	Video question answering	Counterfactual prediction
VC R-CNN [222]	CVPR 2020	Visual common sense, unsupervised learning, feature representation	Visual common sense	Causal intervention
CATT [209]	CVPR 2021	Causal attention module, causal intervention	Visual question answering	Front-door adjustment
- [259]	CVPR 2020	Visual question answering, counterfactual, task analysis, machine learning	Visual question answering	Counterfactual
CF-VQA [258]	CVPR 2021	Computer vision, linguistics, robustness, counterfactual inference	Visual question answering	Counterfactual
CSS [261]	CVPR 2020	Visual-explainable, question-sensitive, counterfactual samples	Visual question answering	Counterfactual
- [260]	CVPR 2020	Semantic editing, robustness, synthetic dataset, data augmentation	Visual question answering	Counterfactual
DeVLBert [262]	ACM 2020	Multi-modal pretraining, out-of-domain, debias, back-door adjustment, BERT	Visual question answering	Back-door adjustment
VLCI [263]	2023	Radiology report generation, visual language pretraining model, cross-modal reasoning	Visual question answering	Front-door adjustment
CMCIR [267]	TPAMI 2023	Visual question answering, cross-modal reasoning, video event understanding	Visual question answering	Front-door adjustment & back-door adjustment
CONTA [46]	NeurIPS 2020	Weakly supervised semantic segmentation, context adjustment, causal inference	Weakly supervised semantic segmentation	Back-door adjustment
C-CAM [228]	CVPR 2022	Weakly supervised semantic segmentation, medical images, class activation mapping	Weakly supervised semantic segmentation	Causal intervention
CF [221]	2021	Zero-shot semantic segmentation, counterfactual, causal inference	Zero-shot semantic segmentation	Counterfactual
- [255]	PRL 2021	Action recognition, causal graph structures, causal relationship, recognition of falls	Action recognition	Causal graph structures
CISNet [256]	AAAI 2022	Causal diagram, subject-invariant facial action unit, causal intervention	Action recognition	Causal intervention
DeCalGAN [220]	TMM 2023	Zero-shot learning, action recognition, causal inference	Zero-shot learning	Causal intervention
- [218]	NIPS 2020	Zero-shot learning, feature compositionality, causal inference	Zero-shot learning	Causal intervention
CaML [219]	NIPS 2024	Meta-learning, causal inference, zero-shot learning	Zero-shot learning	Causal intervention
- [235]	NIPS 2020	Long-tailed classification, back-door adjustment, re-balanced training	Image classification	Causal intervention
TLT [234]	CVF 2023	Noisy image classification, causal inference, attention mechanism	Image classification	Causal intervention
- [232]	ICIP 2021	Visual causality, contrastive explanations, gradients, causal metrics	Image classification	Causal inference

#### Causal inference-based attention model

Attentional modeling is a significant concept in neural networks, with a principle similar to human vision. The model focuses its attention on a specific part, disregarding information in other locations. By filtering out extraneous visual information, the attention model increases visual recognition efficiency, enabling us to better comprehend images, language, and other data. This approach has also been widely applied to neural networks, with a significant impact.

Wang et al. [207] introduced a causal attention module (CaaM) that self-annotates confounders in an unsupervised manner, using causal intervention to eliminate the effects of confounders. The article hypothesizes that attention has opposite effects in IID and OOD tasks, and that attentional modeling is less effective than nonattentional baseline modeling in OOD tasks. The authors attribute this to confounding effects. To eliminate the impact of these confounding factors, they propose a causal intervention approach. Specifically, they constructed a causal graph to describe the relationships between the input image *X*, the label *Y*, the confounder *S*, and the mediating variable *M*. Using a data partitioning intervention method, the training data *T* = {*t*_1_, …, *t_m_*} are partitioned, with each partition representing a confounding layer. A back-door adjustment is employed to cut off the back-door path *X* ← *S* → *Y*. Intervention is performed through data partitioning and iterative self-annotation of confounders. To avoid overfitting, adversarial learning is used to separately learn causal and confounding features. Experimental results show that CaaM achieves more accurate attentional activation than traditional attentional methods. Wang et al. [208] introduced a causal attention network for addressing city-wide spatiotemporal prediction tasks. They introduced a learnable super-region matrix to identify useful correlations between regions while eliminating useless correlations. Through a causal framework, random interventions were generated by collecting useless representations from irrelevant regions. Yang et al. [209] utilized causal models to address the issue of inconsistent data distribution in the training dataset, which cannot be resolved by conventional machine learning models. They proposed a causality-based attention mechanism, which is founded on the principle of front-door adjustment and eliminates the impact of unstable confounders in current attention-based visual language models.

#### Causal inference-based meta-learning

Adapting the learning process dynamically for different tasks is referred to as meta-learning, which relies on past prior knowledge in its training process and is susceptible to the influence of training data. Bengio et al. [210] proposed an intervention-based approach that involves modifying the data distribution to observe potential causal relationships. This essay is to develop a model that can generalize effectively to other datasets with different distributions and establish a robust foundation through prior training. This will enable the model to quickly adapt to new distributions and achieve excellent transfer learning performance. Nair et al. [211] developed an interactive agent for scenario-based learning that possesses causal inference capabilities. The article proposes a 2-stage meta-learning algorithm for the agent. In the first stage, the algorithm discovers causal relationships in raw visual observations, and an attention-based iterative prediction method is used to progressively update the predicted causal graph for each interaction observed in the environment, generalized the causal structure in the form of a DAG. In the second stage, the causal structure is utilized to place the target conditional strategy in context and encode the conditional strategy graph to accomplish the goal-directed task. On the whole, they constructed causal structures by visually observing the environment to achieve goal-directed tasks in a new visual setting. Dasgupta et al. [212] employed model-free reinforcement learning to train neural networks for solving problems that involve causal structures. They trained recurrent neural networks using meta-learning to implement an algorithm capable of performing causal inference.

#### Causal inference-based few-shot learning

Few-shot learning is a critical research area in machine learning, focusing on the development of robust and generalized models with limited labeled data. Its importance stems from addressing the scarcity of labeled data in real-world applications, making it applicable across various tasks including image classification [213], target detection [214,215], and more. The way to achieve fast generalization on small sample data is to rely on prior knowledge. Pretraining is an effective method for acquiring prior knowledge, and a network pretrained on a large dataset can be used for feature extraction. To further optimize the effect of small sample learning, the concept of meta-learning is introduced to train the initial function of the model. However, pretraining can sometimes introduce confounding for model training. Yue et al. [216] conducted an analysis of the causal relationships among features, labels, and pretrained knowledge. They demonstrated that eliminating confusion through causal analysis can help alleviate the negative impact of prior knowledge. To enhance the model’s ability to extract semantic information from limited data, Li et al. [217] introduced causal learning techniques to mitigate the impact of empirical errors from the teacher’s model on the student’s predictive performance in knowledge distillation.

#### Causal inference-based zero-shot learning

Zero-shot learning is a specialized form of few-shot learning that emphasizes the model’s ability to generalize to previously unseen categories. Zero-shot learning typically requires the introduction of additional auxiliary information, such as attribute information, embedding spaces, and cross-domain transfer, to enable the model to apply what it has learned to unknown categories. Atzmon et al. [218] addressed the problem of compositional generalization in zero-shot learning from a causal perspective. They proposed a causal embedding model that considers the generation process or causes of an image as actual objects or labels from the real world. This approach enables the model to better understand the associations between image content and real-world entities, thereby enhancing its ability to recognize and combine information within images.

Nilforoshan et al. [219] proposed a zero-shot causal learning approach for personalized medicine. For each intervention value, *W* is given its specific conditional average treatment effects (CATE) function, which is represented by the following equation:

$$\mathrm{CAT}E_{w}=\tau_{w}\left(x\right)=E_{p}\left[Y\left(w\right)-Y\left(0\right)|X=x\right]$$ (17)

where *X* = *x* denotes an individual characterized by *x*, *Y*(*w*) denotes the outcome under intervention *w*, and *Y*(*0*) as the control state denotes the outcome without the intervention. In this approach, a single meta-model is trained using CATE for each intervention. This enables the model to predict the personalized effects of novel interventions that were not present during the training phase. Wang et al. [220] applied zero-shot learning to video recognition tasks to improve the recognition of unknown action categories. They proposed a new framework called deconfounding causal GAN (DeCalGAN), which includes a reconstruction module and a deconfounding module. By constructing a structured causal graph model, the framework captures the true causal relationships between features and actions, allowing it to infer the feature distribution of unseen categories. Shen et al. [221] employed a counterfactual approach to derive a novel causal intervention map aimed at mitigating the indirect effects of actual features.

#### Causal inference-based visual commonsense learning

Image tasks involving commonsense concepts have gradually attracted attention, which rely more on commonsense knowledge in images or text. When judging the category of the target in the area, we can utilize commonsense contextual visual information to assist in making the judgment. Commonsense information, such as “people walk with their legs”, is challenging to document in text. This kind of commonsense information does not often appear in texts. At the visual level, commonsense information is more apparent. For example, if a table exists in the current image, it is likely that a chair will also be visible. However, these commonly existing commonsense concepts are often difficult to learn because there are other confounders in the image that interfere with the model’s judgment. Wang et al. [222] proposed an unsupervised feature representation method aimed at intervening in visual tasks and discussed learning commonsense knowledge in vision to predict contextual objects under existing labeling conditions. An unsupervised feature representation approach to address observational bias through intervention is proposed. The target features are learned through the intervention *P*(*Y*| *do*(*X*)), which distinguishes between common features and sense-making features, thus eliminating the influence of confounders, reducing the observation bias, and improving the prediction performance of the model.

#### Causal inference-based semantic segmentation

Semantic segmentation is a crucial task in computer vision that aims to identify the semantic category of each pixel in an image, thereby achieving pixel-level understanding. Based on the degree of labeled information available, semantic segmentation can be categorized into fully supervised, semisupervised, weakly supervised, and unsupervised semantic segmentation. Researchers have incorporated causal learning methods into each of these approaches to enhance learning outcomes.

Fully supervised semantic segmentation: Fully supervised semantic segmentation refers to the use of training with explicit pixel-level semantic category labels in the training phase. To help the model better understand the causal relationships between pixels, researchers have introduced causal inference. Li and Li [223] introduced a refined causal segmentation model that employs Transformer as the backbone network. The approach incorporates a causal intervention into the vision transformer and proposes a causal module based on the SCM. Fan et al. [224] applied causal learning to the task of cell nucleus segmentation in the medical field. To enhance the segmentation accuracy of cell nuclei, a simple parameter-free attention module (SimAM) was developed to fuse downsampled features. This module addresses challenges such as edge blurring and spurious noise encountered during the recognition process.

Semisupervised semantic segmentation: Semisupervised semantic segmentation uses a combination of labeled and unlabeled data to train a model to classify each pixel in an image into a semantic category. Miao et al. [225] proposed a novel causal graph, elucidating the importance of algorithm independence in causal learning. Additionally, they designed a min–max optimization process to further enhance the independence of co-training.

Weakly supervised semantic segmentation: Conventional semantic segmentation methods require pixel-level fine labeling, which is a time-consuming and inefficient process. To address this challenge, researchers have proposed weakly supervised semantic segmentation (WSSS), which leverages weak annotations such as image-level labels and bounding boxes to train segmentation models. Notably, WSSS with image-level labels often employs the class activation mapping (CAM) [226,227] approach to extract high-response information through deep convolutional networks. Nevertheless, this localization approach often faces issues such as object foreground–background confusion and severe co-occurrence phenomena. Chen et al. [228] proposed a causal class activation mapping (C-CAM) to investigate the causes of inaccurate activation regions and significant shape variations in WSSS. By constructing 2 causal chains based on category causality and anatomy causality, C-CAM addresses the issue of ambiguous boundaries and co-occurrence phenomena through causal intervention. Generating accurate pseudo-masks is a critical challenge in WSSS because it directly impacts the quality of the final segmentation results. Zhang et al. [46] proposed a framework for improving WSSS through causal inference. They employ an iterative process to generate high-quality pseudo-masks by eliminating confounding factors.

Unsupervised semantic segmentation: Unsupervised semantic segmentation is a method for learning semantic classification of images without any pixel-level annotation, which requires the model to discover the semantic structure in the image. Kim et al. [229] enhanced the performance of unsupervised semantic segmentation by utilizing discrete indices in the mediating variables for self-supervised learning at the conceptual level.

#### Causal inference-based image classification

Image classification [230,231] is a fundamental task in the field of computer vision that aims to assign predefined category labels to the input images. Challenges faced in image classification task include accuracy of feature extraction, image noise and distortion, and category imbalance that can reduce the accuracy of image classification models. To address the above problems, researchers have given solutions from a causal perspective. Prabhushankar and AlRegib [232] proposed a method to extract causal features from the interpretation of image classification networks, using an ensemble theory approach to obtain causal features from Grad-CAM [233] features, thus separating causal features from contextual features. The contextual features are also defined as contrast features, providing an evaluation setup for testing causality in the case of limited labeling. Yang et al. [234] introduced an image classification framework named treatment learning transformer (TLT), applying the concept of causal inference to address image classification in noisy environments. The classification model integrates the notions of confounding factors and do-operators. By combining the conditional variational encoder–decoder (CVED) with attention mechanisms, it effectively models the processing of noisy images, enhancing the classification performance in noisy conditions. Tang et al. [235] analyzed momentum in long-tail classification based on causal learning. Momentum is regarded as a confounding variable, revealing its effects on input features and classification results, and a back-door adjustment method is proposed to eliminate the spurious associations caused by momentum.

#### Causal inference-based object detection

Object detection [236,237] is a crucial task in the field of computer vision, which involves identifying the location of an object by framing it with a target box and assigning it to a specific category. The 2 primary methods used for target detection are single-stage detection based on regression and 2-stage detection based on region suggestion. These methods rely on analyzing correlations between the target instances, bounding boxes, and labels within the dataset. Huang et al. [238] introduced a structured causal intervention module that uses back-door adjustments to cut off causal paths between contextual information and image pixels, thus removing the effects of confounding factors. Zhang et al. [239] proposed a novel domain adaptive model that discovers weather-condition invariant feature representations. The approach utilizes a memory network to store confounders, employs an object detector to extract candidate objects, updates the memory dictionary, and then explores the invariant features of specific objects using a back-door adjustment-based causal inference module. Xu et al. [240] introduced a model termed multi-view adversarial discriminator (MAD), which leverages an autoencoder to map features into different latent spaces and subsequently classifies the transformed features using a multi-view domain classifier. Traditional self-supervised learning-based models for salient object detection often overlook the effects of contrast distribution bias and spatial distribution bias. To address these biases, Lin et al. [241] proposed a de-biasing framework rooted in causal inference, leveraging back-door adjustment to mitigate contrast bias. Additionally, they introduced an image-level weighting method to balance the importance of various spatial locations, effectively alleviating spatial distribution bias.

#### Causal inference-based scenario diagram generation

As research on visual representation progresses, there is a growing demand for images that not only depict the location and category of objects but also the relationships between them. As a result, researchers have turned their attention to developing scene graphs, which provide structured representations of scenes. SGG aims to describe the properties of objects in a scene and their relationships with one another. While common SGG solutions involve detecting object bounding boxes and predicting object classes and pairwise relationships using existing object detectors [242–245], these methods often fail to effectively capture the consistency of the visual context. Most of the unbiased research on SGG tasks has primarily tackled the long-tail problem, neglecting another significant source of bias: semantic confusions induced by textual information. These semantic confusions can lead models to inaccurately predict relationships between variables. Sun et al. [246] addressed both challenges through a causal inference approach. They introduced a 2-stage causal model that accounts for both long-tailed distributions and semantic confusions as confounding factors. Initially, they proposed a novel loss function that leverages statistical insights to penalize the model’s predictions of similarity relations, thereby mitigating the impact of semantic confusion. Then, they introduced an adaptive tuning algorithm aimed at mitigating confusion arising from long-tailed distributions and obtaining unbiased predictions. Chen et al. [247] introduced the counterfactual critic multi-agent training (CMAT) approach. This approach treats the SGG task as a cooperative multi-agent problem to extract counterfactual causality from the trained causal graph and eliminate negative bias. Tang et al. [248] developed a novel framework for SGG based on counterfactual causality. This framework is designed to train unbiased SGG models from biased datasets, thereby eliminating data bias in context (Table 3).

#### Causal inference-based video understanding

The increasing volume of video data has led to a growing interest in spatiotemporal multimodal fusion-based video classification, video action recognition, video action localization, video summarization, and other video understanding tasks.

Video classification: Kanehira et al. [249] proposed a method that generates counterfactual explanations and corresponding spatiotemporal regions for video understanding tasks. It contains a trainable classifier that computes the counterfactuality of a given visual language explanation or region attribute.

Video action localization: Weakly supervised temporal action localization (WTAL) is a crucial method in video tasks that aims to identify the temporal boundaries of an action instance. However, this approach is susceptible to challenges such as classification errors and localization errors, which traditional research has attributed to unlabeled backgrounds. Liu et al. [250] conducted a causal analysis to identify the root causes of problems in WTAL and found that the model results were affected by unobserved confounders in the visual task. Since these confounders cannot be eliminated by data correlation, the paper proposes a temporal smoothing PCA (principal components analysis)-based (TS-PCA) deconfounder that leverages latent variables to generate observed data as a replacement for unobserved data, thereby eliminating confounding effects. Nan et al. [251] presented a causal-based solution to the video localization problem, which involved identifying a specific moment within a video based on a given textual query.

Video summarization: The goal of video summarization tasks is to condense videos in a way that effectively communicates the overall story while retaining essential information. Huang et al. [252] proposed a causal explanation model called Causalainer. A causal semantic extractor is constructed using transformer blocks to extract essential features from multi-modal inputs and perform feature concatenation.

Video action recognition: Video action recognition [253,254] is also one of the key tasks in video understanding, aiming at recognizing the actions of people or objects in a video. Action recognition requires analyzing the content of each image frame in a video and mining the associations between video frames from timing information. In order to better understand the biases in action recognition systems and the risks they pose, and to enhance the credibility of the models, researchers have introduced causal inference to help build action recognition models. Lai et al. [255] devised a system-level architecture for fall action detection, integrating a causal network with a fall detector. The causal network captures biases affecting fall detection performance, such as subject attributes like weight, gender, and age. Subsequently, a BN is constructed to delineate the causal relationships among these attributes. Chen et al. [256] analyzed facial images, subjects, latent action unit (AU) semantic relations, and estimated AU occurrence probabilities by constructing a causal graph. The effects from confounding factors were eliminated using a back-door adjustment method.

#### Causal inference-based VQA

The training of VQA [257] models is often hindered by linguistic bias, which impedes their ability to learn the complex relationships between vision and language. Models that rely solely on memorizing strong linguistic priors from the training data are unlikely to generalize well. Consequently, it is crucial to remove the effect of linguistic bias from the training process to enhance the performance of the VQA models.

Image-based VQA: Niu et al. [258] proposed a method to mitigate linguistic bias in VQA by treating it as a direct causal effect of questions on answers. They subtracted the direct linguistic effect from the total causal effect. Abbasnejad et al. [259] proposed a framework that utilizes counterfactuals to anticipate interventions on the input and trains the model with a set of imaginary alternative samples to learn responses. Agarwal et al. [260] leveraged model-independent counterfactual samples for training. They masked the essential components of the image and question to generate counterfactual samples and discerned the informative features in the dataset by training on these counterfactual samples. Chen et al. [261] proposed a method for synthesizing counterfactual samples for training by obscuring key objects and important words in the original images to generate counterfactual image data and questions. Zhang et al. [262] utilized causal theory to analyze the objective function of BERT [263] pretraining and identify factors that may result in data bias. The proposed intervention-based BERT architecture generates multiple intervention-based de-biased conditional prediction modules to replace or enhance masked language modeling (MLM) objectives. In the medical domain, to address the challenges faced by AI-based radiology report generation (RRG), Chen et al. [263] constructed an SCM to identify the causal effects between vision and language using front-door causal intervention. By employing language mediation, the back-door path from confounders to the outcome is eliminated, resulting in deconfounded visual-linguistic features. This approach helps reduce the disparity between generated medical reports and real reports.

Video-based VQA: VQA tasks are not only based on images but also on videos. Video question answering is a task that involves understanding text based on video content, which requires capturing relationships between multimodal data. Zang et al. [100] proposed a multimodal causal reasoning (MCR) framework from the perspective of causal learning. The framework utilizes causal intervention to separate causal features and confounding factors from visual and textual information. Based on word encoding and the relevance between words and text, important keywords that significantly impact prediction results are selected. These keywords are then combined with other candidate answers to form negative samples, thereby improving the model’s recognition capability. Li et al. [264] proposed the context-aware video intent reasoning model (CaVIR), which incorporates a multi-head self-attention module to integrate all contextual features and obtain a composite feature representation of the video. Wei et al. [265] constructed SCMs and combined a front-door intervention method with question semantics to extract representative video clips and frames, forming visual causal scenes crucial for generating dependable answers. Chen et al. [266] employed self-supervised local autoregression to train the model, thereby circumventing the requirement for question–answer (Q&A) pairs during training and diminishing the dependence on data annotation. The training process of the model involves leveraging a learned generative model to analyze the test data and identify changing factors from the typical causal process. Counterfactual conditions are introduced to substitute the original conditional variables, enabling predictions of potential alterations. To address spurious correlations between cross-modal variables, Liu et al. [267] proposed a cross-modal causal inference and reasoning (CMCIR) framework. The framework consists of a vision-language reasoning module that relies on visual perception. It includes a visual causal module based on front-door intervention, using attention mechanisms to aggregate local and global visual representations. Additionally, it includes a language causal module based on back-door intervention, approximating the distribution of confounding factors from a semantic perspective. It utilizes a spatiotemporal transformer (STT) to model multimodal interactions between appearance-motion and language representations, and eliminates language biases based on an SCM.

## Causal Datasets

In this section, we present datasets commonly utilized for causal inference tasks, as listed in Table 4. It is worth mentioning that in some tasks, such as visual representation tasks, it may be necessary to directly evaluate the effects of causal-based models on traditional benchmarks rather than on specific causal datasets.

**Table 4.** General overview of datasets commonly used for various causal tasks

Types of causal	Dataset	Year	Data sources	Task	Key characteristics
Causal effect	e-CARE [271]	2022	Human annotated	Natural language processing	Text reasoning, sentence level labeling, commonsense causal inference benchmark
	ATOMIC [272]	2019	Human annotated	Natural language processing	Text reasoning, generative training, if–then relation types
	CaTeRS [273]	2016	Text generation	Natural language processing	Temporal relation, causal semantic, sentence level labeling
	ESC v0.9 [274]	2017	Human annotated	Natural language processing	Event storyline corpus, causal relation extraction, timeline
	BECauSE 2.0 [275]	2017	Human annotated	Natural language processing	Causal language, causal relationship, co-present semantic relations
	COCO-QA [279]	2015	Real images/Q&A generation	Visual language task	Image-based question-answering, question-answer generation
	CLEVR [280]	2017	Synthetic images/Q&A generation	Visual language task	Image-based question-answering, visual reasoning capabilities of VQA models, image generation, question generation
	CLEVRER [281]	2019	Synthetic video/Q&A generation	Visual language task	Video representation and reasoning, temporal relation
	VCR [282]	2019	Movie scenes/Q&A generation	Visual language task	Visual commonsense reasoning, adversarial matching, explanatory questions
	Visual Genome [283]	2017	Real images/human annotated	Visual language task	Relationships, scene graph, region descriptions, question answer pairs
	VQA v1.0 [284]	2015	Real images/human annotated	Visual language task	Visual question answering, open-ended questions & answers
	VQA v2.0 [285]	2017	Real images/human annotated	Visual language task	Visual question answering, open-ended questions & answers, complementary images
	TVQA [286]	2018	Movie videos/human annotated	Visual language task	Timestamp annotation, video question answering
	SpatialVLM [287]	2024	Real images/Q&A generation	Visual language task	Spatial reasoning, automatic 3D spatial VQA data generation framework
	VSR [288]	2023	Real images/Q&A generation	Visual language task	Spatial reasoning, template-based caption generation
	Social-IQ [289]	2019	Real videos/human annotated	Visual language task	Socially intelligent, rigorous annotation, open-ended questions and answers
	CUVA [290]	2024	Real videos/human annotated	Anomaly understanding	Causation understanding, video anomaly
Causal relations	MIMIC II/III Data [291,292]	2002/2016	Real data	Data statistic	Time-stamped, critical care information, causal effect
	Geo Experiment Data [293]	2017	Synthetic data	Data statistic	Estimating causal effects, counterfactual time series, geo-based
	Twins [294]	2017	Real data	Data statistic	Infant health, interventions, causal effect
	Air Quality Data [295]	2011	Real data	Data statistic	Air quality, air concentrations of ozone, time-stamped
	Economic Data for Spanish Regions [296]	2003	Real data	Data statistic	Terrorist conflict, causal effect

### Causal datasets for NLP

Researchers have become increasingly aware of the commonsense errors caused by dataset bias in NLP tasks. Most models are trained on the characteristics of only one or a few datasets, which limits their ability to identify commonalities across languages and hinders their reasoning power. Therefore, understanding the interrelationships between data through causal inference is an effective way to correct prediction bias. Initially, machine learning-based methods, such as using random forest algorithms to explore interactions in high-dimensional data, were used to explore causal relationships in the data. Later, deep learning models were introduced, and many text-based causal inference datasets have also been proposed.

e-CARE (https://github.com/Waste-Wood/e-CARE/files/8242580/e-CARE.zip): The e-CARE dataset [268] is a manually annotated explainable causal inference dataset that contains over 21,000 causal inference problems. For each problem, a natural language description of the conceptual explanation of why the current causal relationship can hold is also provided.

ATOMIC (https://maartensap.com/atomic/data/atomic https://huggingface.co/datasets/allenai/atomic): The ATOMIC dataset [269] is a valuable resource for commonsense inference based on if–then relations. For instance, in the statement “*X* repelled *Y*’s attack”, humans can easily deduce various outcomes, such as *X*’s motivation for the action, *X*’s character traits, and the potential effects on both *X* and *Y*. However, computers currently lack the ability to make such inferences. The ATOMIC dataset addresses this gap by proposing 9 hypotheses based on 3 if–then relations and collecting over 877,000 instances of inferred knowledge. By utilizing generative training, the model can acquire a basic understanding of commonsense reasoning.

CaTeRS (http://cs.rochester.edu/nlp/rocstories/CaTeRS/): A semantic annotation framework was introduced by [270] to capture temporal and causal relationships in stories. This framework was applied to annotate 1,600 sentences from 320 short stories, revealing the causality and temporal relationships among them. Furthermore, numerous causal inference corpora based on text have been proposed, such as phrases [271,272] and sentences [273], which have been extracted and annotated from large open domain web text corpora [195,269,274,275].

### Causal datasets for visual language tasks

Humans possess the remarkable cognitive ability to combine temporality and causality in images or videos to describe ongoing events or predict their future direction. To train machines to have similar capabilities, researchers have proposed several causal datasets that train models to reason about causal relationships in events and explore the logical relationships behind the reasoning. These datasets are based on static [276,277] or dynamic [278–280] forms, which challenge the model’s recognition ability and primarily focus on enhancing its capacity to reason about events and make counterfactual predictions.

CUVA (https://github.com/fesvhtr/CUVA): As a comprehensive benchmark for causal understanding of video anomaly, CUVA [281] contains 1,000 high-quality annotations for real-world videos, including 10 major anomaly types and 42 sub-anomaly types. The benchmark describes in detail why anomaly occur and their corresponding effects through manual annotation methods. The average length of the benchmark videos is 117 s, covering 4.3 sentences.

COCO-QA (https://www.cs.toronto.edu/mren/research/imageqa/data/cocoqa/): This dataset [282] consists of images and annotations derived from the MS-COCO dataset. Automated generation of Q&A pairs for each image is based on the annotations in MS-COCO, assuming that responses can only consist of a single word. This dataset comprises 123,287 images, of which 78,736 were used for training and 38,948 for testing.

CLEVR (https://cs.stanford.edu/people/jcjohns/clevr/): In [283], a dataset called CLEVR was created to explore the visual inference capacity of VQA models through linguistic and visual inference. This dataset comprises 100,000 simple 3D images and 1 million automatically generated problems related to counting, inference, and other areas. The recognition function is deliberately weakened to intensively train the model’s inference ability. The dataset includes objects with 3 shapes, 2 materials, and 8 colors. These objects are annotated with information about their properties, location, and other relevant details.

CLEVRER (http://clevrer.csail.mit.edu/): Expanding on the image properties of the CLEVR dataset, researchers in [284] introduced the CLEVRER dataset, which is based on videos and collision-based inference. This dataset comprises 20,000 synthetic videos of colliding objects and more than 300,000 questions and answers generated by a physics engine simulating object motion and a graphics engine rendering frames. The dataset is divided into 10,000 videos for training, 5,000 for validation, and 5,000 for testing, with each video lasting 5 s. The objects in the videos use intrinsic properties of composition similar to CLEVR, including 3 shapes, 2 materials, and 8 colors. The question categories in the dataset include descriptive, explanatory, predictive, and counterfactual questions.

VCR (https://visualcommonsense.com/download/): To facilitate visual commonsense reasoning, Zellers et al. [285] introduced the VCR dataset, which includes screenshots of ongoing events from 110,000 movies. Adversarial matching is used to generate nontrivial and high-quality questions on a large scale, with 24% being descriptive, 38% explanatory, and 13% predictive, requiring inferences about future events. To mitigate the impact of statistical bias in the dataset on the model and eliminate misleading a priori knowledge in the real world, the paper proposes a new dataset for visual inference and question answering. This dataset includes 22 million inference questions constructed using Visual Genome Scene Graphs [285].

Visual Genome (https://homes.cs.washington.edu/ranjay/visualgenome/api.html): The Visual Genome dataset [286], which comprises 108,000 images, is an invaluable resource for advancing cognitive development and improving image interpretation accuracy. Each image contains an average of 35 objects, 26 attributes, and 21 pairs of relationships between objects. The dataset’s primary focus is on labeling relationships between objects, making it an essential tool for facilitating visual commonsense reasoning.

VQA v1.0 (https://visualqa.org/download.html): VQA v1.0 [276] used images from the COCO dataset [282], with 123,287 images used for training and 81,434 images used for testing. Additionally, the dataset included 614,163 questions from human annotations. Open-ended questions and answers are given for different regions of the image, including foreground information and background details. Nevertheless, the inherent linguistic bias can influence the outcomes.

VQA v2.0 (https://visualqa.org/download.html): The updated version in [287], which is built upon version 1.0, utilizes the MS COCO dataset [288]. Additional images were gathered to complement the existing ones, and a second round of data annotation was performed on these new images to obtain new answers. This approach resulted in a larger dataset with a more homogeneous distribution, reducing the impact of data bias. The dataset includes 1.1 million image–question pairs and 13 million answers, with 443,757 image pairs in the training set, 214,354 image pairs in the validation set, and 447,793 image pairs in the test set.

TVQA (https://tvqa.cs.unc.edu/download tvqa.html): Lei et al. [289] constructed a large-scale video Q&A dataset derived from 6 classic American dramas, with a total of 21,800 video segments of 60 to 90 s in duration. The dataset contains 152,500 manually labeled Q&A pairs with temporal localization.

Social-IQ (https://github.com/A2Zadeh/Social-IQ): The Social-IQ dataset [278] is proposed to train and evaluate the social skills of intelligent systems and construct explanatory social intelligence. This dataset is complexly and rigorously annotated for each video scenario and contains 1,250 social scenarios in natural environments, 7,500 questions, and a total of 52,500 answers (30,000 correct answers and 22,500 incorrect answers).

SpatialVLM (https://spatial-vlm.github.io/): Chen et al. [290] has developed an automated framework for generating 3D spatial VQA data that creates a large-scale dataset containing 10 million images and 2 billion direct spatial reasoning Q&A pairs.

VSR (github.com/cambridgeltl/visual-spatial-reasoning): Liu et al. [291] believed that current visual language models struggle to capture spatial relationships; thus, they developed the VSR dataset. This dataset consists of over 10,000 natural text–image pairs, containing a total of 66 spatial relationships such as *under*, *in front of*, and *facing*.

VQAI (https://github.com/IEIT-AGI/MIX-Shannon/blob/main/projects/VQAI/lgd_vqai.md): Li et al. [292] proposed a new image generation task called visual question answering with image (VQAI). Based on the classic Tom and Jerry cartoon series, a dataset of the same name has been established. A total of 755 Tom and Jerry cartoons produced between 1940 and 2021 have been divided into smaller video clips, each containing a single story plot. The annotator extracts a pair of causally related images from each segment and annotates the pair with questions. The average length of the questions is 18.1 words, and most image inferences can be completed in 1 to 2 steps.

### Effect inference datasets

We discuss some real-world datasets commonly used for effect inference tasks.

MIMIC II/III Data (https://physionet.org/content/mimiciii/1.4/): The dataset [293,294] captures various data from the monitor of a critically ill patient, including physiological signals, vital sign time series, input medications, intake fluids (solution, blood), and output fluids (urine, blood). This comprehensive set of data can be utilized in various predictive or counterfactual causal inference tasks [295,296].

Geo experiment data: In a similar vein, Kerman et al. [297] developed a time-based regression (TBR) method to analyze geological experiments. This method is capable of predicting counterfactual time series and evaluating cumulative causal effects.

Twins: Louizos et al. [298] comprised data on twins born in the United States between 1989 and 1991, encompassing their gestation time, prenatal care, and the mother’s underlying physical condition.

Air quality data (https://www.aeaweb.org/articles?id=10.1257/aer.101.6.2687): Auffhammer and Kellogg [299] investigated the impact of gasoline content regulations on ozone pollution in the United States. The dataset included ozone concentrations, daily minimum and maximum temperatures, as well as rainfall and snowfall data from 1989 to 2003. The time series data in the dataset enable the exploration of causal effects between different variables.

Economic data for Spanish regions (https://www.jstor.org/stable/3132164): Abadie and Gardeazabal [300] studied the impact of terrorist conflicts on the economy of the region. They utilized terrorism activity data provided by the Spanish Ministry of the Interior, as well as regional data such as gross domestic product (GDP), investment, and population density, to draw causal inferences about the impact of terrorism on the economy. These data do not have a ground truth value.

### Causal discovery datasets

We discuss some real-world datasets commonly used for causal discovery tasks.

Human motion capture: This dataset comprises motion trajectories of human bodies in 3D space, including information such as body positions and joint angles of subjects at 2,024 time points. Tank et al. [301] applied this dataset to a causality discovery task.

U.S. manufacturing growth data: The dataset comprises microeconomic data on the growth rates of employment, sales, research and development expenditures, and operating income in the U.S. manufacturing sector from 1973 to 2004. Due to the fact that most companies do not report their data every year, the dataset is somewhat unbalanced. Entner and Hoyer [302] and Moneta et al. [303] used this dataset to infer causal relationships between variables.

Diabetes dataset: The dataset consists of identity information and physiological data, including various blood test results and hormone levels, of 442 patients, along with their disease progression after 1 year. Schaechtle et al. [304] applied his proposed multidimensional causal discovery method to study causal relationships among variables in this dataset.

## Open Questions

Causal inference, initially a philosophical concept that defined a particular characteristic of human cognition, has been integrated into science. Scientists have developed a mathematical language that figuratively expresses the causal relationship between various entities and have used this language to simulate the logical thinking of causal inference on modern computers. The notion of causal inference is now widely applied in various fields to advance the development of machine intelligence. Moving forward, we outline the top 10 research areas and challenges that require focused attention in advancing causal inference.

Limitations of causal inference: We discuss some of the current limitations of causal inference methods.

1. Distinguishing between correlation and causation among variables is challenging in real-world tasks: Confounders can be identified in common tasks by constructing causal graphs [163,166], generative hypotheses [167,173], and other methods. However, distinguishing whether variables are correlated or causal in the real world is difficult due to the inherent complexity and numerous uncertainties of real-world causal relationships. These uncertainties include randomness, hidden variables, and potential confounders, which, if not properly accounted for and excluded, can affect the model’s judgment.

2. Lack of publicly available benchmarking resources to train and evaluate causal models: Current causal-based deep learning often suffers from a lack of publicly available benchmark resources for training and evaluating causal models. Real-world interventions are challenging and expensive to conduct, making it difficult to create these resources. To better assess the effectiveness of causal models, we need publicly available benchmark intervention datasets and counterfactual datasets, which are currently scarce. Cheng et al. [305] argue that acquiring intervention data, which requires active interactions with the environment, is more challenging than obtaining data from passive observations. Accessing real-world counterfactual instances is even more impractical. This scarcity of benchmark datasets has slowed progress in CDL [69].

3. Current work lacks comparison with equally causal-based approaches: Most current research claims that incorporating causal learning improves model performance compared to noncausal methods. However, these studies typically compare their results only with noncausal approaches and not with other causally based methods [44,217,306]. We believe that comparing with other causally based methods can better validate the proposed methods and prevent overestimation or misleading conclusions about their performance. Such comparisons can also foster further development in the field of causality, advancing the technology and enhancing our ability to solve complex problems.

4. It is necessary to raise the awareness of causal learning among researchers in related fields: The value of causal inference in deep learning is still being explored and validated. It is hoped that increasing research in this area will facilitate the widespread application of causal inference across various domains. This will attract more researchers to develop advanced and innovative methods and techniques, thereby advancing the field of causal inference.

Causal inference in AI: As AI continues to advance, the question arises: Can we create advanced intelligence that can truly think? Currently, computer models provide answers by identifying patterns rather than achieving genuine comprehension. It is like giving a child an encyclopedia and a question—they may not comprehend the question or the contents of the book, but they can search, compare, and analyze similar questions and corresponding answers to arrive at an answer. Similarly, computers cannot fully grasp what they have learned, but they can calculate answers based on patterns they have identified. However, this falls short of the desired outcome as people expect AI to have the ability to reason. As causal inference develops and becomes integrated into various fields, there will be a growing focus on research into advanced intelligence that can truly think.

The next step in the development of AI: Researchers have classified the development of AI into 3 stages: weak AI, strong AI, and super AI. Weak AI is applied AI designed to solve specific tasks in specific fields, encompassing all AI currently in use. Strong AI, on the other hand, is a general-purpose AI that can perform all the tasks that humans can perform. However, we have not yet reached the level of strong AI, which requires a mature AI that can learn, think, and communicate like a human being. Super AI, which surpasses human intelligence and computing power, is capable of performing tasks beyond human ability. While some believe that super AI will eventually surpass human intelligence, others argue that the current level of AI development is far from achieving this goal.

Brain cognition in machine reasoning: Traditional artificial neural networks are based on the concepts and structures of neurons and synapses in the human brain’s nervous system. This allows for the hierarchical information processing mechanisms of the brain to be mimicked, providing AI with a model for information processing. Furthermore, the reasoning and attention mechanisms of the human brain can effectively address numerous fundamental issues in AI. While the current understanding of the cognitive, reasoning, and attention mechanisms of the human brain has helped neural network models achieve success in many fields, further research into the principles and mechanisms of brain cognition is essential to attain higher levels of intelligence. Deeper exploration of these brain mechanisms will also unlock new opportunities for the development of AI.

Data distribution and recommender systems combined with inference: One of the major limitations of AI lies in its reliance on IID data. Traditional recommendation systems assume that the dataset meets the IID criteria during both the training and testing phases. However, in reality, most application data for recommendation systems is out of distribution, which significantly impacts the effectiveness of the models. There are 2 main causes of OOD in recommender systems. The first is a natural shift, a migration that occurs in natural dimensions such as time and space. The second type is artificial shift, in which the recommender system trains the model based on observed data. The results obtained from the feedback interact with the user by providing information, and new observed data are obtained after the interaction. If the model is trained based solely on correlation, it will be influenced by the data and unable to reason out the underlying reasons. Therefore, it is essential to develop new techniques that can handle OOD data and improve the robustness and generalization ability of recommendation systems.

Physics-informed causal inference: There are many strict causal relationships in physics, such as the causal relationship between the initial state and the interacting forces in a mechanical system. This relationship reflects the established laws that govern the objective world and the unity of chance and necessity that exist in causality. Additionally, the complex and regular motion in the entire mechanical system is also a result of causal relationships. Causality is a pervasive concept throughout the entire physical system, and it has a rich theoretical basis in the fields of optics and quantum science. In recent years, there has been a growing interest in physics-informed machine learning. This approach aims to merge physical priors and data to improve the effectiveness of machine learning models. The integration of mathematical and physical models with data can offer dependable solutions for machine learning tasks and enhance model performance [307].

Data-independent causal models: The current linguistic and visual causal datasets are prone to data bias, which can result in erroneous model assessments and adverse consequences. To mitigate the impact of statistical bias in the dataset, future research should focus on developing data-independent models based on causal inference to reduce the reliance of causal models on data.

Causality assessment: Currently, there is inconsistency in assessing causal effects due to variations in data and models. Therefore, a more authoritative and rational framework for causal assessment is imperative at this stage.

Interpretability of causal learning: Interpretability is a crucial attribute that helps in comprehending a model’s decision-making principles. However, achieving transparent interpretability in highly complex deep models remains a daunting task. Causal inference can effectively address this aspect. Causal relationships are not mere correlations; they are more strictly causal, which makes them more stable than correlations and less susceptible to interference in multivariate tasks. Hence, applying causal inference to various domains to enhance interpretability is an important research area for scholars.

The impact of AI with reasoning ability: If AI possesses reasoning ability, it has the potential to process vast amounts of data and information more accurately and efficiently, leading to more intelligent decisions and services, ultimately improving human work efficiency. However, it may also have negative consequences, such as inference errors, machine reasoning results that do not meet human ethical and moral standards, excessive reliance on machine reasoning leading to reduced human thinking ability, and encroachment of machine intelligence on human job opportunities. This implies that the development of AI reasoning must anticipate potential issues, devise corresponding measures to prevent or minimize negative impacts, and enable AI’s reasoning ability to create positive impacts and transformations in human society.

## Conclusions

With the development of big data, causal inference has been widely studied in many data-driven fields such as statistics, physics, and computer science. In recent years, causal inference has also gradually become a direction of interest in the field of deep learning, which can provide an effective approach to reveal causal relationships between variables in realistic tasks and improve the interpretability and robustness of models. The purpose of this survey is to provide a comprehensive, detailed overview of the concepts and applications of causal inference in the field of deep learning.

In this paper, we review the fundamental concepts and common approaches in causality research, differentiating between 2 main directions in causal learning: causal inference (understanding causal effects) and causal discovery (identifying relationships in data), along with related research efforts. We analyze research ideas in reasoning from a brain cognition perspective, discussing the necessity and limitations of causal inference. Additionally, the mathematical formulas and common reasoning frameworks for causal inference are summarized. The reasoning capabilities of large models and the contribution of causal inference to enhancing these models are also examined. The cross-study of causal inference methods in classical algorithms for deep learning is discussed. Most importantly, we provide a comprehensive overview of causal inference applications in deep learning tasks across various data modalities: voice (speech processing), text (NLP), graphics (graph representation learning), and images (visual representation). It is hoped that this will deepen the reader’s understanding of causal applications in real-world tasks to advance their research. Common datasets used for causal learning, along with their data properties and corresponding download links, are summarized. Finally, the top 10 open problems in causal inference are given, and the limitations of current methods as well as future research directions are discussed.
